# Heart Rate Variability and Direct Current Measurement Characteristics in Professional Mixed Martial Arts Athletes

**DOI:** 10.3390/sports8080109

**Published:** 2020-07-30

**Authors:** Joseph O. C. Coyne, Aaron J. Coutts, Roman Fomin, Duncan N. French, Robert U. Newton, G. Gregory Haff

**Affiliations:** 1 School of Medical and Health Sciences, Edith Cowan University, Joondalup, WA 6027, Australia; dfrench@ufc.com (D.N.F.); r.newton@ecu.edu.au (R.U.N.); g.haff@ecu.edu.au (G.G.H.); 2 UFC Performance Institute, Las Vegas, NV 89118, USA; rfomin@ufc.com; 3 Human Performance Research Centre, University of Technology Sydney, Moore Park Rd, Moore Park, NSW 2021, Australia; Aaron.Coutts@uts.edu.au; 4 School of Sport, Exercise and Rehabilitation, University of Technology Sydney (UTS), Moore Park Rd, Moore Park, NSW 2021, Australia; 5 School of Behavioral and Health Sciences, Australia Catholic University, Fitzroy, VIC 3065, Australia; 6 Directorate of Physiotherapy and Sport, University of Salford, Salford, Greater Manchester M6 6PU, UK

**Keywords:** Omegawave, acute recovery and stress scale, measurement characteristics

## Abstract

This study’s purpose was to examine heart rate variability (HRV) and direct current potential (DC) measures’ sensitivity and correlations between changes in the acute recovery and stress scale (ARSS) and the previous day’s training load. Training load, HRV, DC and ARSS data were collected from fourteen professional mixed martial arts athletes (32.6 ± 5.3 years, 174.8 ± 8.8 cm, 79.2 ± 17.5 kg) the following morning after hard, easy and rest days. Sensitivity was expressed as a signal-to-noise ratio (SNR, inter-day typical error (TE) or coefficient of variation (%CV) divided by intra-day TE or %CV). Correlations between HRV, DC and ARSS with training load were also examined. The SNRs for the various HRV and DC measures were acceptable to good (1.02–2.85). There was a 23.1% CV average increase between measures taken between different locations versus the same location. Training load changes were not correlated with HRV/DC but were correlated with ARSS stress variables. Practitioners should be aware of HRV/DC variability; however the daily training signal was greater than the test-retest error in this investigation. Upon awakening, HRV/DC measures appear superior for standardization and planning. HRV and DC measures were less sensitive to the previous day’s training load than ARSS measures.

## 1. Introduction

Various monitoring tools are applied in professional sports to help estimate an athlete’s potential risk of injury and readiness to train or compete [1,2]. The information provided by these tools may be used to facilitate decision-making regarding of planning of training [2]. Important aspects of choosing any athlete monitoring tool include the reliability and sensitivity of that particular tool [3,4]. Reliability measures of athlete monitoring tools can be measured using a test-retest analysis by taking repeated measures from the same individual under as close as possible to identical test conditions [5,6]. This approach can be used to calculate reliability statistics including an intra-class correlation (ICC), an absolute typical error (TE), a standardised typical error (sTE) and a coefficient of variation (%CV). A signal-to-noise ratio (SNR) can also be calculated to help determine the sensitivity of any test and is considered valuable for determining the utility of a measurement device [6,7,8]. In professional sport, the measurement signal is commonly the variation in athlete test results in response to training (e.g., inter-day or inter-week %CV or TE) whereas the measurement noise is the typical error of the test (e.g., intra-day %CV or TE) [7,8].

One of the most popular objective athlete monitoring tools is heart rate variability (HRV) [9]. HRV is the variability between successive heart beats (R–R interval) and is considered a non-invasive indicator of the status of the cardiovascular branch of the autonomic nervous system [10]. The status of the autonomic nervous system may be of interest for coaches and athletes as it has been suggested to be useful for monitoring an athletes’ readiness to train, long term training adaptations and for optimising training prescriptions [11,12]. Portable short-duration HRV assessment methods of ~1–5 min have been demonstrated to be effective [11,13]. However, factors such as the choice of HRV measures (e.g., root mean square of sum of differences (RMSSD)), measurement position (e.g., supine, seated, standing), water intake prior to measurement, sex, time of measure (e.g., nocturnal, upon awakening, upon arrival at training or after submaximal exercises) and breathing rate may all affect HRV measures [11,14,15,16]. Of particular interest for coaches is if there are differences between HRV measures taken upon awakening (which are recommended as best practice when using heart rate measures to guide training prescription) [17] and measures taken upon arrival at training (which may be preferred by coaches for compliance reasons) [18]. Previous research by Sherman [18] on collegiate female rowers measuring HRV (RMSSD) at these two time-points/locations suggests that either time point/location can be used, although the upon awakening measurements were more correlated with performance measures. It is unknown if this remains the case for male athletes and/or professional athletes.

Another objective athlete monitoring tool that has been far less researched compared to HRV is direct current potential (DC). Direct current potential is defined as very slow brainwave activity (0–0.5 Hz) and can be measured through electrodes placed on the scalp or a combination of forehead and thenar eminence [19,20,21]. The DC has been suggested to reflect the state of the athlete’s CNS [22,23,24] and also appears to be correlated with electroencephalography measures [21]. Both DC and HRV seemingly provide different information and there is a potential for autonomic nervous system status to be practically unrelated to CNS status in an athlete in some cases [25]. Further, although autonomic nervous system measures seem to be important for most sports, they may not be as relevant as CNS measures for certain power-orientated sports like weightlifting or sprinting [25].

For these reasons and for practitioners wishing to monitor both HRV and DC with their athletes, of interest is the commercially available Omegawave^®^ system. The Omegawave^®^ system is a portable short duration (~5 min) measurement device using a smartphone/tablet application, Bluetooth sensor, heart rate and electrocardiography chest strap and electrodes [22,24,26,27]. The Omegawave^®^ system provides a number of measures for analysis that are derived from HRV and DC recordings [22,24,27]. The main outputs of the Omegawave^®^ system that practitioners will normally use when employing the device with athletes are the proprietary readiness and Windows of Trainability™ measures [27]. The proprietary readiness measures are overall readiness, cardiac readiness and CNS readiness [27]. Meanwhile, the Windows of Trainability™ are divided into endurance, skill, speed and power, and strength readiness [27]. Despite the growing popularity of the Omegawave^®^ system for professional athletes [22,24,26], the authors were only able to find one investigation examining the critical measurement properties of the Omegawave^®^ system’s raw DC measures [21] with no research seemingly available examining the HRV, proprietary readiness and Windows of Trainability™ measures.

Alongside objective athlete monitoring tools like HRV and DC, there is also considerable interest in subjective athlete monitoring tools [25,28]. Comparing objective and subjective tools, a recent systematic review of the research showed that subjective measurements of athlete readiness like psychometrics were more sensitive and consistent to changes in training load than objective measures like HRV [28]. However, DC was not one of the objective tool assessed in this systematic review [28] and the authors could not locate any research examining the relationship between DC and subjective athlete readiness measures. Although subjective measures may be preferred for their sensitivity to training load and low cost, objective measures like HRV may also be better suited to assessing medium- to long-term training adaptation [12]. Another consideration is that, despite being less related to training loads than subjective measures, HRV or other objective measures of readiness may be more related to performance outcomes. As such, practitioners and coaches are recommended to use both subjective and objective measures to gain a better understanding of athlete readiness [25,29].

Due to the popularity of the Omegawave^®^ system, combined with the apparent lack of research on its critical measurement properties, the first purpose of this study was to investigate the intra-day reliability and sensitivity of the HRV and DC measures from the Omegawave^®^ system in professional athletes. The second purpose was to examine the Omegawave^®^ system’s different HRV/DC measures for intra-day reliability and sensitivity at two different time-points/locations (upon awakening and upon arrival at training location). This was considered important given the practical importance for coaches deciding how to use the Omegawave^®^ system and the current lack of research examining the difference between HRV measures at different time-points/locations on the same day in professional athletes. Finally, the correlations between changes in the previous day’s training load, a subjective athlete readiness measure, and the different Omegawave^®^ HRV/DC measurements were examined to better understand subjective and objective athlete-readiness measurements’ relationship with training load. This would also seem to be the first investigation examining DC’s relationship with subjective measurements in professional athletes.

## 2. Materials and Methods

### 2.1. Experimental Approach to the Problem

This observational investigation consisted of four analyses; (i) test-retest reliability at the same location, (ii) signal-to-noise ratio, (iii) test-retest reliability at different time-points/locations, and (iv) correlations between changes in HRV and DC, a subjective measure of athlete readiness (the Acute Recovery and Stress Scale (ARSS) [30]) and the previous day’s training load as training impulse (TRIMP). All HRV and DC data were collected and analysed using the Omegawave^®^ device (Omegawave Oy, Espoo, Finland). The HRV, DC, ARSS and TRIMP measures were collected in the morning on the day after a hard (+1HARD), easy–moderate (+1EASY) and rest (+1REST) day in the same training week. As the participants were not all coached by the same mixed martial arts coach, classification of the hard, easy–moderate and rest days was primarily determined by the athletes’ individual coaches and were quantified with TRIMP scores to confirm the athletes’ relative training load.

### 2.2. Subjects

Fourteen professional mixed martial arts male (n = 10) and female (n = 4) athletes (33 ± 5 years, 174.8 ± 8.8 cm, 79.2 ± 17.5 kg) participated in this investigation. All athletes were currently signed to the Ultimate Fighting Championship’s or another mixed martial arts promotion’s roster. All athletes were also without serious injury and were required to participate fully in coach-planned training during the investigation. Written informed consent was given by all participants and approval for this investigation was granted by the University Human Ethics Committee (Approval #19521) and conforms to the Code of Ethics of the World Medical Association (Declaration of Helsinki).

### 2.3. Procedures

For all analyses, no interventions or modifications of training, diet or recovery based on the observed HRV/DC, ARSS or TRIMP data were made during the data collection period. All participants undertook familiarisation procedures with the Omegawave^®^ device, ARSS and TRIMP data collection procedures and requirements prior to commencing the actual measurement trials. Each individual HRV and DC measurement took ~5 min and were all completed in a quiet air-conditioned room or section of the training location for that day (~21–23 °C). All HRV and DC measurements were standardised for temperature (as above), measurement position and breathing rate, i.e., completed supine with arms by the participants’ side (as per Omegawave^®^ recommendations), and were instructed to inhale for 5 s and exhale for 5 s during data collection. This breathing rate was considered ecological for professional athletes in a supine relaxed position fell within “autonomically optimised respiration” [31] and ensured consistent breathing rates intra- and inter-day; which was important to standardise considering the potential influence breathing rate has on some HRV variables, e.g., frequency-domain variables [14,16]. Participants maintained their normal training, diet, recovery and lifestyle practices with the exception of standardising water intake as much as possible (which was recorded for later analysis) and were asked to avoid consuming caffeine prior to any HRV/DC measurements.

#### 2.3.1. Test-Retest Reliability at the Same Location

Five minutes after arrival at the training location for the first scheduled training session on +1HARD, +1EASY or +1REST mornings, participants completed three consecutive HRV/DC measurements (i.e., nine total HRV/DC recordings over the three days). The %CV or TE, ICC and sTE were then calculated between the three measures for each of +1HARD, +1EASY or +1REST respectively and not over all three days combined. Each measurement was separated by 5 min. During this time, participants were asked to repeat the conditions prior to the first measure to ensure similar postural effects on blood pressure and heart rate and other stimulation effects from interaction with other participants or electronics (e.g., smartphones).

#### 2.3.2. Signal-To-Noise Ratio

The participants’ HRV and DC measurement signal was defined as the inter-day %CV or TE between +1HARD, +1EASY and +1REST. The inter-day %CV or TE was calculated from the first measures of each day. The measurement noise was defined as the average intra-day %CV or TE over the three data collection days (i.e., the average of the +1HARD%CV/TE, +1EASY%CV/TE and +1REST%CV/TE; not the %CV over the three days). The measurement signal was then divided by the measurement noise to compute the SNR [7,8].

#### 2.3.3. Test-Retest Reliability at the Different Time-Points/Locations

Participants completed two HRV and DC measurements on three different days: an initial measurement 15–30 min after awakening, that was self-administered, and another measurement upon arrival at their first morning training session of the day at the participants’ respective training locations. The amount of time between measures ranged from 1–3 h in the morning depending on athletes’ training schedules. The %CV/TE, ICC and sTE were calculated between the two measures.

#### 2.3.4. Correlations between Changes in HRV and DC Variables, ARSS Variables and the Previous Day’s TRIMP

TRIMP scores were recorded from participants for their training sessions on the day prior to +1HARD, +1EASY and +1REST. The TRIMP scores were calculated by multiplying the participants’ session ratings of perceived exertion (sRPE) with session duration, as described by Foster et al. [32] The previous day’s TRIMP was used in light of research showing that the sensitivity of athlete readiness measures do not seem to be improved when accounting for training loads beyond the previous day [33]. Participants also completed the ARSS on each of the three data collection days before collecting the HRV and DC measures. The ARSS was recorded using a customised online form (Google Forms, Google LLC, Mountain View, CA, USA) and has been validated for English speakers and consists of eight components (i.e., physical performance capacity, mental performance capacity, emotional balance, overall recovery, muscular stress, lack of activation, negative emotional state, overall stress) [30]. Following the collection of the ARSS on each individual day, participants then began the HRV and DC measurement process. Changes in TRIMP scores from the day prior to +1HARD, +1EASY and +1REST were then correlated using repeated measure correlations to both the changes in ARSS components and the changes in selected HRV and DC measures.

### 2.4. Statistical Analyses

Statistical analyses were performed using statistical software (R statistics package, https://www.r-project.org, version 3.6.3, R Foundation for Statistical Computing, Vienna, Austria) or purposefully designed Excel spreadsheets (Microsoft Corporation, Washington, DC, USA) [34]. The full R console code has been provided in the Supplementary Material for reference. All data are presented as mean ± standard deviation. The alpha level for significance for all tests was defined as *p* ≤ 0.05. A sample size of n = 10 was considered adequate from power calculations prior to the investigation for the statistical tests with a β of 0.80 [35] and an anticipated ICC of 0.90. Variance in the measures were assessed using the F-test and for normality using the Shapiro–Wilk test and visual inspection of quantile-quantile (Q-Q) and density plots. Depending on distribution and variance, either an independent *t*-test (student’s or Welch’s) or Mann–Whitney U test were applied. Effect sizes (Hedge’s g) with 95% confidence intervals were then calculated between the +1HARD, +1EASY and +1REST. Effect sizes were interpreted as per the recommendations of Hopkins et al. [5] For the reliability statistics, TE, %CV, ICC and sTE along with their 95% confidence intervals were calculated using Hopkins’ recommendations [36,37]. All the raw HRV and DC measures were log-transformed and analysed as %CV to account for any sex differences in raw measures [36,37]. Meanwhile, the Omegawave^®^ proprietary readiness and Windows of Trainability™ measures, which are on an arbitrary 1–7 or 1–4 scale, were assessed as the raw TE [36,37]. The sTE were calculated to provide practitioners with an alternative analysis of typical error beyond %CV and ICC. Interpretation of sTE are the same as the effect size interpretations mentioned above [5]. From the TE and %CV, the minimal detectable change based on a 95% limit of agreement (MDC_95_) was also calculated [38]. The classification of SNRs were adapted from previous research [7,8]. SNRs were classed as poor if ≤1, acceptable if >1 and good if the signal did not fall below the upper 95% confidence interval of the noise and the noise did not fall above the bottom 95% confidence interval of the signal (similar to a one-tailed t-test with *p* < 0.05). Repeated measures correlations with 95% confidence intervals were then calculated on the changes between HRV/DC variables, ARSS components and the previous day’s TRIMP. Similar to effect size magnitudes, correlations were interpreted as per the recommendations of Hopkins et al. [5]

## 3. Results

The participants’ descriptive statistics of HRV/DC data from +1HARD, +1EASY and +1REST are presented in Table 1. Descriptive statistics of the participants’ TRIMP and ARSS data are also presented in Table 2. There were significant differences in TRIMP between all three days (*p* < 0.01, *g* = 1.12–3.90). For the HRV/DC measures, there were significant differences in DC and CNS Readiness between +1REST to +1EASY (*p* = 0.01 and 0.02, *g* = 0.54 and 0.52) and +1REST to +1HARD (*p* = 0.01 and 0.04, *g* = 0.56 and 0.45). There was also a significant difference between +1EASY to +1HARD in the Overall Recovery dimension of the ARSS (*p* = 0.03, *g* = 0.87). There was no significant difference between water consumption on the three different days prior to HRV/DC measurement (*p* = 0.85, 0.27 and 0.28).

**Figure 1:**
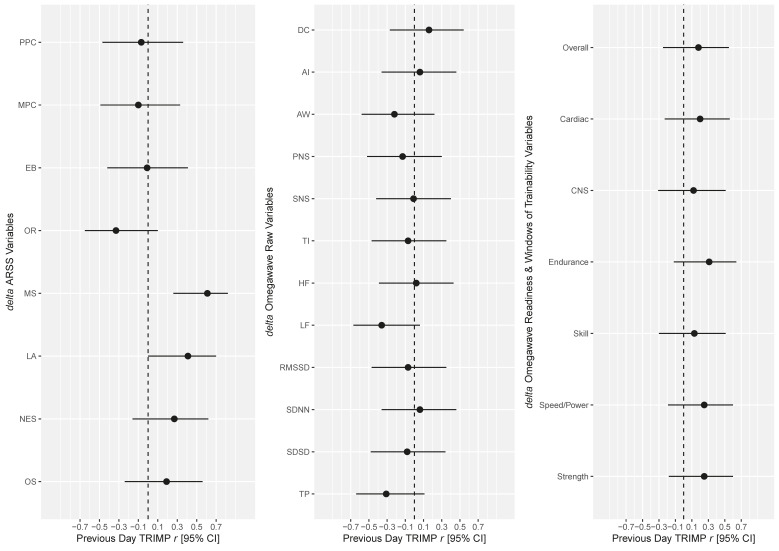
The correlation between changes in ARSS variables and changes in Omegawave^®^ heart rate variability and direct current potential variables with the previous day’s training impulse. TRIMP—training impulse; ARSS—Acute Recovery and Stress Scale; PPC—physical performance capacity; MPC—mental performance capacity; EB—emotional balance; OR—overall recovery; MS—muscular stress; LA—lack of activation, NES—negative emotional state; OS—overall stress; DC—direct current; AI—aperiodic influences; AW—aspirate waves; PNS—parasympathetic activity; SNS—sympathetic activity; TI—Tension; HF—high frequency; LF—low frequency; RMSSD—root mean square of sum of differences; SDNN—standard deviation of NN interval; SDSD—standard deviation of differences between NN intervals; TP—total power; CNS—central nervous system, *r*—correlation; CI—confidence interval.

The data from the intra-day test-retest reliability analysis at the same time-point/location is provided in Table 3. The SNRs for the various HRV/DC measures are listed in Table 4. The SNRs ranged from acceptable to good. The test-retest reliability analysis between HRV/DC measures at different time-points/locations (i.e., upon awakening at home and upon arrival at the training location) are presented in Table 5. There was an average increase of 23.1% in intra-day %CV at different time-points/locations compared to intra-day %CV at the same time-point/location. Finally, the repeated measures correlations between changes in the previous day’s TRIMP, ARSS and HRV/DC Omegawave^®^ measures are displayed in Figure 1. The previous day’s TRIMP was significantly correlated with two ARSS components—muscular stress (*r* = 0.61, *p* = 0.001) and lack of activation (*r* = 0.41, *p* = 0.04). There were no significant correlations with the previous day’s TRIMP in any of the raw HRV/DC measures or Omegawave^®^’s proprietary readiness and Window of Trainability™ measures.

## 4. Discussion

The key finding of this investigation is that there are differing levels of reliability and sensitivity in the HRV/DC measures from the Omegawave^®^ system in professional athletes. There were also large differences between measures taken upon awakening and measures taken upon arrival at the training location in this investigation. Lastly, the subjective measure of readiness (ARSS) in this investigation were more related to the previous day’s TRIMP than the HRV and DC variables.

There were differing levels of reliability and sensitivity in the HRV/DC measures from the Omegawave^®^ system seen in this investigation. The authors have presented three main methods of interpreting reliability (%CV, ICC and sTE) along with the MDC_95_ to provide a more comprehensive approach in evaluating the reliability of each measure. Of note is that the reliability of DC in this investigation seemed lower than noted in Valenzuela et al.’s previous investigation (ICC 0.97 (0.93, 0.99)) [21]. This may be due to subject characteristics (i.e., professional mixed martial arts athletes compared to healthy individuals), the greater number of repeated measures in this investigation and the timing of measurements in each of the investigations (e.g., upon awakening may be better suited to measure DC for reliability purposes). Moreover, of particular interest are the proprietary Omegawave^®^ readiness and Windows of Trainability™ measures which, as mentioned previously, are the main outputs of the Omegawave^®^ system that practitioners would normally use when employing the device with athletes. The overall cardiac and CNS readiness measures had TE that ranged from 0.60–1.10 on a 1–7 scale, ICC from 0.57–0.78 and sTE from 0.47–0.56 (moderate). The Endurance, Skill, Speed and Power and Strength Windows of Trainability™ measures had TE that ranged from 0.52–1.22 on a 1–4 scale, ICC from 0.51–0.96 and sTE from 0.46–0.71 (moderate to large). Based upon the data in this study, the authors recommend practitioners use caution when interpreting both the raw HRV/DC data and the readiness/Windows of Trainability™ variables that the Omegawave^®^ system provides. Future research using the Omegawave^®^ system or any other HRV/DC measurement devices should also be wary of the (perhaps inherent) levels of variability in these measures.

The authors also recommend that practitioners interpret any measurement reliability data in light of the typical signal, i.e., the change that occurs under typical training conditions. For instance, a measurement device may have a high amount of noise (e.g., high intraday %CV) but if the signal significantly and consistently exceeds that noise, then this device may be more useful in identifying changes in athletes than a measurement device that has a low amount of noise but the signal does not significantly exceed that noise. In this case, we have provided the HRV and DC measures’ SNR to aid in this evaluation of sensitivity and overall usefulness of these measures for informing decisions on training availability and optimal training choices. In the raw measures, aspirate waves, DC potential, metabolic reaction index, root mean square of sum of differences (RMSSD), standard deviation of NN interval (SDNN), standard deviation of differences between NN intervals (SDSD) and tension index all had good SNRs (1.74–2.85). In support of recommendations for RMSSD to be the primary HRV variable of choice for practitioners [9], the SNR for RMSSD was the second highest (2.82) behind only SDSD. The remaining raw measures’ SNR were acceptable (1.02–1.48). All of the proprietary Omegawave^®^ readiness and Windows of Trainability™ measures possessed acceptable-to-good SNRs (1.06–1.71). From these data, it seems HRV and DC measures’ signals after different training days (e.g., hard, easy, rest) exceeds any noise in professional mixed martial arts athletes. This implies that the HRV/DC measures were sufficiently sensitive to detect physiological change from training and is a promising finding for practitioners wishing to use the Omegawave^®^ system to objectively monitor athlete readiness. It is currently unknown how the Omegawave^®^ system calculates readiness and Windows of Trainability™ measures and whether they use smoothing methods (e.g., weekly rolling averages) or single data points for raw variable analysis. If smoothing methods are not currently employed, doing so may also potentially improve the measurement intraday reliability and decrease noise in the SNR [9,12]. The authors also suggest that any future measurement device SNR be interpreted using confidence intervals in preference over arbitrary ranges (e.g., an SNR above 1.5 is good) [39].

The next part of this investigation was an examination of the difference between HRV and DC measures taken upon awakening or prior to the first training session of the day. Overall, there was ~23% greater %CV between different location repeated measures compared to the same location repeated measures (as in the first part of this study). Of note is the DC potential %CV difference (97.2%) and the very large sTE between measurements taken at the different locations. What is also worthy of mention is that the difference in RMSSD %CV (10.8%) between locations was much larger than presented in Sherman’s previous research on this topic [18]. This may be related to the differing time lengths between measurement collection at the different time-points/locations in each respective investigation (i.e., within one hour [18] compared to up to 3 h in this investigation). However, similar to Sherman [18], this investigation and the DC reliability data in Valenzuela et al. [21] supports the recommendation that HRV and DC measures should be taken immediately after awakening. This would allow for greater standardization and lower influence of confounding factors like meals, travel and stimulants on any measures pre-training [17]. Measures taken first thing in the morning also give coaches and practitioners more time to decide how to modify training and recovery plans (if needed) before the first training session of the day, which is an important logistical consideration [9].

The final aspect of this study concerned the relationships between the previous day’s TRIMP, the ARSS and the HRV/DC measures. In this group of professional athletes, the ARSS was more sensitive to the previous day’s TRIMP than the HRV/DC measures. This was highlighted by the significant correlations in changes in previous day’s TRIMP, with changes in muscular stress and lack of activation. The results agree with Saw et al. [28], who concluded that subjective measures were more sensitive and consistent to changes in training load than objective measures like HRV. Although practitioners are encouraged to employ both subjective and objective markers of internal load, it seems prudent for subjective measures to influence *acute* decision making on athlete readiness to a greater extent than objective measures [25,28] and subjective measures may be needed to help contextualize changes in HRV/DC when deciding training modifications on a short-term basis [9]. However, it is worth considering that both measures may demonstrate different recovery trends (e.g., HRV may recover faster than subjective measures [40]) and objective measures may be better suited to monitor medium- to long-term training adaptation [12]. Furthermore, although subjective measures currently seem more sensitive to training load than objective measures, this may be different in respect to other important variables like performance.

A potential limitation of this investigation is that both reliability and sensitivity were evaluated “in the field” and not in a strictly controlled environment like a laboratory. Although this may have led to higher %CV between the repeated measures, the reliability results of this investigation can be interpreted as a “worst-case” scenario, it is an ecologically valid representation of the environments in which athlete monitoring tools are often used. Another limiting factor was that this group of professional athletes were all from the same sport (mixed martial arts) and these results may not be applicable to other sports. It should also be noted that this study was of a relatively short time frame (i.e., one training week) and investigations into longer durations may be worthwhile if an assessment of longer term training adaptations is wanted.

## 5. Conclusions

Despite the differences in reliability measures, all HRV and DC variables measured by the Omegawave^®^ device were found to have acceptable-to-good SNR. This suggests these HRV and DC measures have acceptable sensitivity to different training days (e.g., hard, easy, rest) in professional athletes. We also found large differences in measures taken upon awakening and measures taken upon arrival at the training location. It seems that HRV and DC measures taken upon awakening are superior if able to be practically implemented. Finally, changes in the professional athletes’ HRV and DC variables did not present the same level of sensitivity to changes in previous day training load when compared to the ARSS, a subjective measure of athlete readiness.

If choosing to use the Omegawave^®^ or other HRV/DC measurement devices, practitioners should be aware of the variability in these measures. Despite this variability, it seems that the SNR of the various measures from the Omegawave^®^ device are acceptable. To improve the measurement precision, practitioners are recommended to control food, caffeine and water intake prior to measurement. If practitioners choose to extract them, reliability may also be potentially enhanced by applying smoothing methods to the raw variables, e.g., a 3–7 day simple or exponentially weighted moving average. It seems HRV/DC measures taken upon awakening are preferred for standardization and planning purposes (e.g., having more time to adjust training if needed based on results). Furthermore, practitioners are encouraged to utilise both HRV/DC and subjective measures like the ARSS to assess athlete readiness to train or compete. For acute decisions on athlete readiness, this combination may need to be weighted towards subjective measures given the higher correlations in changes in ARSS with previous training loads.

## Figures and Tables

**Table 1:** Descriptive statistics for heart rate variability and direct current potential variables measured using the Omegawave system in professional mixed martial arts athletes after three different training days.

	+1REST (1)	+1EASY (2)	+1HARD (3)	*p*			*g*		
				1–2	1–3	2–3	1–2	1–3	2–3
Raw Variables									
Aperiodic Influences (s)	2.2 ± 0.8	2.1 ± 0.7	2.2 ± 0.9	0.43	0.57	0.91	0.17	0.12	0.02
Aspirate Waves (AU)	0.06 ± 0.04	0.06 ± 0.03	0.05 ± 0.03	0.51	0.10	0.27	0.14	0.35	0.24
DC Potential (mV)	14.9 ± 12.4	8.9 ± 9.6	15.3 ± 13.0	0.01 *	0.86	0.01 **	0.54	0.04	0.56
HF (ms^2^)	396 ± 325	355 ± 327	438.9 ± 428.7	0.68	0.70	0.28	0.08	0.08	0.22
LF (ms^2^)	1588 ± 1675	1280 ± 1117	1185.6 ± 1100.5	0.91	0.67	0.61	0.02	0.10	0.10
LF/HF	6.2 ± 7.6	5.5 ± 4.7	5.0 ± 6.4	0.65	0.32	0.11	0.10	0.22	0.32
MRI	213.6 ± 73.8	221.7 ± 96.1	219.2 ± 71.2	0.69	0.46	0.88	0.08	0.16	0.04
PNS (s)	0.6 ± 0.2	0.6 ± 0.2	0.6 ± 0.2	0.28	0.35	0.95	0.24	0.20	0.01
RMSSD (ms)	77.4 ± 42.9	72.1 ± 31.3	73.7 ± 34.3	0.51	0.65	0.82	0.14	0.10	0.05
SDNN (ms)	98.1 ± 43.4	88.0 ± 34.2	91.3 ± 38.9	0.29	0.56	0.67	0.22	0.12	0.09
SDSD (ms)	97.1 ± 53.6	90.8 ± 38.4	92.5 ± 43.1	0.54	0.66	0.84	0.13	0.09	0.04
SNS (%)	0.4 ± 0.1	0.5 ± 0.1	0.5 ± 0.1	0.07	0.09	0.97	0.39	0.18	0.00
Tension (AU)	78.1 ± 119.5	87.9 ± 180.6	156.9 ± 477.8	0.11	0.19	0.90	0.34	0.28	0.02
Total Power (ms^2^)	2099 ± 1823	1731 ± 1219	1758.4 ± 1236.5	0.65	0.70	0.99	0.10	0.08	0.00
Scale Variables									
Overall Readiness (1–7)	5.5 ± 1.5	5.6 ± 1.4	5.7 ± 1.6	0.84	0.49	0.27	0.04	0.14	0.22
Cardiac Readiness (1–7)	5.9 ± 1.6	6.3 ± 1.4	6.1 ± 1.7	0.14	0.30	0.66	0.32	0.22	0.10
CNS Readiness (1–7)	5.8 ± 1.1	5.3 ± 1.0	5.8 ± 0.9	0.02 *	0.54	0.04 *	0.52	0.12	0.45
Endurance WOT (1–4)	2.4 ± 0.8	2.5 ± 0.6	2.6 ± 0.8	0.90	0.11	0.08	0.02	0.35	0.37
Skill WOT (1–4)	2.2 ± 0.7	2.0 ± 0.9	2.1 ± 0.9	0.39	0.95	0.44	0.18	0.02	0.16
Speed/Power WOT (1–4)	1.6 ± 1.3	1.4 ± 1.3	1.9 ± 1.1	0.71	0.35	0.19	0.08	0.20	0.28
Strength WOT (1–4)	2.2 ± 0.9	2.2 ± 0.7	2.3 ± 0.9	0.81	0.65	0.41	0.06	0.10	0.18

DC—direct current; HF—high frequency, LF—low frequency; MRI—metabolic reaction index; PNS—parasympathetic activity; RMSSD—root mean square of sum of differences; SDNN—standard deviation of NN interval; SDSD—standard deviation of differences between NN intervals; SNS—sympathetic activity; WOT—Windows of Trainability™; AU—arbitrary units; s—seconds; ms—milliseconds, mV—millivolt; +1REST—measure the day after rest day; +1EASY—measures the day after easy–moderate training day; +1HARD—measures the day after hard training day; *g*—Hedge’s *g* effect size; * *p* < 0.05; ** *p* < 0.01.

**Table 2:** Descriptive statistics for subjective training impulse and Acute Recovery and Stress Scale dimensions in professional mixed martial arts athletes after three training days of different intensity.

	+1REST (1)	+1EASY (2)	+1HARD (3)	*p*			*g*		
				1–2	1–3	2–3	1–2	1–3	2–3
Total TRIMP (AU)	0 ± 0	520 ± 244	890 ± 383	0.00 ***	0.00 ***	0.01 **	3.90	3.90	1.12
ARSS Recovery									
PPC	4.1 ± 1.4	4.3 ± 1.1	4.1 ± 1.2	0.69	0.92	0.74	0.16	0.04	0.13
MPC	4.4 ± 0.9	4.4 ± 0.9	4.3 ± 1.1	0.85	0.87	0.74	0.07	0.06	0.13
EB	4.5 ± 1.4	4.5 ± 1.0	4.5 ± 1.1	0.90	0.97	0.92	0.05	0.02	0.04
OR	3.5 ± 1.5	3.9 ± 1.1	3.2 ± 1.2	0.76	0.15	0.03 *	0.12	0.61	0.87
ARSS Stress									
MS	2.3 ± 1.4	2.8 ± 1.0	3.2 ± 1.2	0.26	0.08	0.40	0.45	0.70	0.33
LA	1.6 ± 1.2	1.7 ± 1.4	2.2 ± 1.2	0.96	0.25	0.31	0.02	0.46	0.39
NES	1.3 ± 1.4	1.5 ± 1.3	1.5 ± 1.4	0.55	0.51	0.94	0.24	0.26	0.03
OS	2.4 ± 1.7	2.3 ± 1.3	2.4 ± 1.4	0.91	0.98	0.91	0.05	0.01	0.04

TRIMP—training impulse; ARSS—Acute Recovery and Stress Scale; PPC—physical performance capacity; MPC—mental performance capacity; EB—emotional balance; OR—overall recovery; MS—muscular stress; LA—lack of activation, NES—negative emotional state; OS—overall stress, +1REST—measure the day after rest day; +1EASY—measures the day after easy–moderate training day; +1HARD—measures the day after hard training day; *g*—Hedge’s *g* effect size; * *p* < 0.05; ** *p* < 0.01; *** *p* < 0.001.

**Table 3:** Intraday test-retest reliability for heart rate variability and direct current potential variables measured using the Omegawave system at same location in professional mixed martial arts athletes after three training days of different intensity.

	+1REST		+1EASY		+1HARD		OVERALL			
Raw Variables										
	%CV	ICC	%CV	ICC	%CV	ICC	%CV (95% CI)	ICC (95% CI)	sTE (95% CI)	MDC_95_
Aperiodic Influences	37.5	0.12	29.8	0.51	40.0	0.20	35.9 (26.2, 56.7)	0.28 (−0.12, 0.67)	0.86 (0.65, 1.26)	99.4
Aspirate Waves	29.6	0.92	28.9	0.91	44.0	0.84	34.7 (25.4, 54.8)	0.89 (0.73, 0.96)	0.36 (0.28, 0.53)	96.1
DC Potential	25.1	0.82	32.5	0.82	35.5	0.69	31.4 (23.0, 49.2)	0.80 (0.54, 0.93)	0.49 (0.37, 0.71)	87.0
HF	79.8	0.78	53.5	0.85	81.2	0.80	71.7 (50.7, 121)	0.81 (0.56, 0.93)	0.48 (0.36, 0.70)	199
LF	46.8	0.93	43.2	0.90	83.6	0.87	63.2 (45.0, 105)	0.90 (0.74, 0.97)	0.35 (0.27, 0.52)	175
LF/HF	89.6	0.82	67.7	0.78	88.6	0.77	82.0 (57.5, 140)	0.80 (0.54, 0.93)	0.48 (0.37, 0.71)	227
MRI	7.70	0.96	4.60	0.99	5.26	0.97	6.05 (4.56, 8.99)	0.98 (0.93, 0.99)	0.17 (0.13, 0.25)	16.8
PNS	17.1	0.89	16.9	0.86	25.4	0.81	20.1 (14.9, 30.8)	0.85 (0.64, 0.95)	0.42 (0.32, 0.62)	55.7
RMSSD	15.3	0.97	13.9	0.97	20.1	0.96	16.6 (12.4, 25.3)	0.96 (0.90, 0.99)	0.21 (0.16, 0.31)	46.0
SDNN	14.4	0.95	15.0	0.94	21.1	0.93	17.1 (12.7, 26.0)	0.94 (0.85, 0.98)	0.27 (0.20, 0.40)	47.4
SDSD	14.7	0.97	13.3	0.97	19.7	0.96	16.1 (12.0, 24.5)	0.96 (0.91, 0.99)	0.21 (0.16, 0.31)	44.6
SNS	12.7	0.86	7.76	0.93	12.9	0.85	11.3 (8.5, 17.0)	0.88 (0.70, 0.96)	0.38 (0.29, 0.56)	31.3
Tension	41.8	0.92	25.8	0.95	52.9	0.89	41.0 (29.8, 65.5)	0.92 (0.79, 0.97)	0.32 (0.24, 0.47)	114
Total Power	47.5	0.92	34.0	0.92	67.1	0.86	50.5 (36.4, 82.1)	0.90 (0.74, 0.96)	0.35 (0.27, 0.52)	140
Scale Variables										
	TE	ICC	TE	ICC	TE	ICC	TE (95% CI)	ICC (95% CI)	sTE (95% CI)	MDC_95_
Overall Readiness (1–7)	1.10	0.45	0.46	0.91	0.67	0.85	0.78 (0.59, 1.14)	0.75 (0.46, 0.91)	0.53 (0.40, 0.78)	2.16
Cardiac Readiness (1–7)	1.10	0.52	0.46	0.92	0.44	0.94	0.72 (0.55, 1.06)	0.81 (0.57, 0.93)	0.47 (0.35, 0.68)	1.99
CNS Readiness (1–7)	0.60	0.67	0.46	0.81	0.62	0.58	0.57 (0.43, 0.83)	0.72 (0.41, 0.90)	0.56 (0.43, 0.82)	1.58
Endurance WOT (1–4)	0.75	0.07	0.32	0.75	0.36	0.82	0.51 (0.38, 0.74)	0.55 (0.16, 0.82)	0.70 (0.53, 1.02)	1.41
Skill WOT (1–4)	0.52	0.40	0.35	0.86	0.49	0.70	0.46 (0.35, 0.67)	0.71 (0.39, 0.89)	0.57 (0.43, 0.83)	1.27
Speed and Power WOT (1–4)	1.22	0.05	0.75	0.72	0.87	0.49	0.96 (0.73, 1.41)	0.46 (0.05, 0.77)	0.76 (0.58, 1.11)	2.67
Strength WOT (1–4)	0.69	0.32	0.20	0.93	0.39	0.81	0.47 (0.35, 0.68)	0.68 (0.34, 0.88)	0.60 (0.46, 0.88)	1.30

DC—direct current; HF—high frequency, LF—low frequency; MRI—metabolic reaction index; PNS—parasympathetic activity; RMSSD—root mean square of sum of differences; SDNN—standard deviation of NN interval; SDSD—standard deviation of differences between NN intervals; SNS—sympathetic activity; WOT—Windows of Trainability™; +1REST—measure the day after rest day; +1MOD—measures the day after moderate training day; +1HARD—measures the day after hard training day; %CV—percent coefficient of variation; ICC—intraclass correlation; TE—typical error, sTE—standardised typical error, MDC_95_—minimal detectable change based on 95% limit of agreement.

**Table 4:** The signal-to-noise ratios of heart rate variability and direct current potential variables measured using the Omegawave system in professional mixed martial arts athletes.

Raw Variables				
	Inter-Day %CV (95% CI)	Intra-Day %CV (95% CI)	SNR	Interpretation
Aperiodic Influences	36.5 (26.3, 59.2)	35.9 (26.2, 56.7)	1.02	Acceptable
Aspirate Waves	76.9 (53.5, 135)	34.7 (25.4, 54.8)	2.21 *	Good
DC Potential	68.2 (47.8, 118)	31.4 (23.0, 49.2)	2.17 *	Good
HF	91.4 (62.9, 164)	71.7 (50.7, 121)	1.28	Acceptable
LF	86.2 (59.5, 153)	63.2 (45.0, 105)	1.36	Acceptable
LF/HF	116 (78.2, 216)	82.0 (57.5, 140)	1.41	Acceptable
MRI	13.3 (9.84, 20.6)	6.05 (4.56, 8.99)	2.20 *	Good
PNS	27.3 (19.9, 43.5)	20.1 (14.9, 30.8)	1.36	Acceptable
RMSSD	46.9 (33.5, 77.7)	16.6 (12.4, 25.3)	2.82 *	Good
SDNN	38.9 (28.0, 63.4)	17.1 (12.7, 26.0)	2.27 *	Good
SDSD	46.0 (32.9, 76.2)	16.1 (12.0, 24.5)	2.85 *	Good
SNS	16.8 (12.4, 26.1)	11.3 (8.5, 17.0)	1.48	Acceptable
Tension	71.3 (49.8, 124)	41.0 (29.8, 65.5)	1.74 *	Good
Total Power	71.0 (49.6, 123]	50.5 (36.4, 82.1)	1.41	Acceptable
Scale Variables				
	**Inter-day TE (95% CI** **)**	**Intra-day TE** **(** **95% CI** **)**	**SNR**	**Interpretation**
Overall Readiness (1–7)	1.25 (0.94, 1.86)	0.78 (0.59, 1.14)	1.60 *	Good
Cardiac Readiness (1–7)	1.24 (0.93, 1.85)	0.72 (0.55, 1.06)	1.71 *	Good
CNS Readiness (1–7)	0.90 (0.68, 1.34)	0.57 (0.43, 0.83)	1.59 *	Good
Endurance WOT (1–4)	0.53 (0.40, 0.80)	0.51 (0.38, 0.74)	1.06	Acceptable
Skill WOT (1–4)	0.73 (0.55, 1.09)	0.46 (0.35, 0.67)	1.58 *	Good
Speed and Power WOT (1–4)	1.13 (0.85, 1.69)	0.96 (0.73, 1.41)	1.18	Acceptable
Strength WOT (1–4)	0.70 (0.53, 1.05)	0.47 (0.35, 0.68)	1.51 *	Good

DC—direct current; HF—high frequency, LF—low frequency; MRI—metabolic reaction index; PNS—parasympathetic activity; RMSSD—root mean square of sum of differences; SDNN—standard deviation of NN interval; SDSD—standard deviation of differences between NN intervals; SNS—sympathetic activity; WOT—Windows of Trainability™; %CV—percent coefficient of variation; SNR—signal-to-noise ratio; 95%CI—95% confidence interval, * *p* < 0.05.

**Table 5:** Intraday test-retest reliability for heart rate variability and direct current potential variables measured using the Omegawave system between time upon awakening at home and prior to training at the training location in professional mixed martial arts athletes.

Raw Variables				
	%CV (95% CI)	Difference %CV	ICC (95% CI)	sTE (95% CI)
Aperiodic Influences	43.2 (31.4, 69.0)	7.4	0.18 (−0.13, 0.51)	2.23 (1.70, 3.26)
Aspirate Waves	49.9 (36.0, 80.6)	15.1	0.85 (0.67, 0.94)	0.44 (0.34, 0.65)
DC Potential	129 (87.6, 235)	97.2	−0.06 (−0.29, 0.27]	>4.0
HF	93.7 (63.3, 163)	22.0	0.79 (0.56, 0.91)	0.55 (0.42, 0.80)
LF	105 (72.3, 184)	41.3	0.80 (0.57, 0.91)	0.53 (0.40, 0.78)
LF/HF	79.6 (56.1, 135)	−2.4	0.84 (0.65, 0.93)	0.46 (0.35, 0.67)
MRI	67.8 (48.3, 113)	61.8	0.32 (−0.02, 0.63)	1.51 (1.15, 2.21)
PNS	21.3 (15.8, 32.6)	1.2	0.82 (0.62, 0.92)	0.49 (0.37, 0.71)
RMSSD	27.4 (20.2, 42.4)	10.8	0.91 (0.79, 0.96)	0.34 (0.26, 0.49)
SDNN	26.9 (19.8, 41.6)	9.8	0.81 (0.60, 0.92)	0.50 (0.38, 0.74)
SDSD	25.5 (18.9, 39.4)	9.4	0.92 (0.82, 0.97)	0.31 (0.23, 0.45)
SNS	13.9 (10.4, 21.0)	2.6	0.80 (0.58, 0.91)	0.52 (0.40, 0.76)
Tension	51.0 (36.8, 82.5)	10.0	0.87 (0.70, 0.94)	0.42 (0.32, 0.61)
Total Power	87.8 (61.5, 151)	37.2	0.80 (0.58, 0.91)	0.53 (0.40, 0.77)
Scale Variables				
	**TE (95% CI)**	**Difference TE**	**ICC (95% CI)**	**sTE (95% CI)**
Overall Readiness (1–7)	0.94 (0.72, 1.38)	0.16	0.73 (0.56, 0.92)	0.65 (0.49, 0.94)
Cardiac Readiness (1–7)	0.80 (0.61, 1.17]	0.08	0.82 (0.71, 0.95)	0.49 (0.37, 0.71)
CNS Readiness (1–7)	1.10 (0.84, 1.61]	0.54	−0.12 (−0.58, 0.26)	>4.0
Endurance WOT (1–4)	0.35 (0.26, 0.51)	−0.16	0.78 (0.65, 0.93)	0.56 (0.42, 0.81)
Skill WOT (1–4)	0.74 (0.56, 1.08)	0.28	0.32 (−0.03, 0.72)	1.52 (1.16, 2.22)
Speed and Power WOT (1–4)	0.98 (0.75, 1.43)	0.02	0.37 (0.04, 0.75)	1.25 (1.03, 1.98)
Strength WOT (1–4)	0.57 (0.44, 0.84)	0.11	0.63 (0.42, 0.88)	0.80 (0.61, 1.17)

DC—direct current; HF—high frequency, LF—low frequency; MRI—metabolic reaction index; PNS—parasympathetic activity; RMSSD—root mean square of sum of differences; SDNN—standard deviation of NN interval; SDSD—standard deviation of differences between NN intervals; SNS—sympathetic activity; WOT—Windows of Trainability™; %CV—percent coefficient of variation; 95%CI—95% confidence interval; Difference %CV—difference in CV% between different location and same location; ICC—intraclass correlation; sTE—standardised typical error, INTERP—interpretation.

## Supplementary Materials

The following are available online at https://www.mdpi.com/2075-4663/8/8/109/s1, code S1: OW Rel Supplement.
