# Supplementary material for: Heart Rate Variability and Direct Current Measurement Characteristics in Professional Mixed Martial Arts Athletes
Source: Sports (Basel). 2020 Jul 30;8(8):109. doi: 10.3390/sports8080109 (PMC7466710; doi:10.3390/sports8080109)
Supplement: Supplementary file 1 [file sports-08-00109-s001.pdf]

sports

Supplementary Materials

Heart Rate Variability and Direct Current Measurement Characteristics in Professional Mixed Martial Arts Athletes

Joseph O.C. Coyne [1,2,*], Aaron J. Coutts [3,4], Roman Fomin [2], Duncan N. French [1,2,5], Robert U. Newton [1] and G. Gregory Haff [1,6]

code S1: OW Rel Supplement.

```
#load required packages
library(tidyverse)
library(dplyr)
library(ggplot2)
library(cocor)
library(corrplot)
library(corrr)
library(car)
library(dabestr)
library(GGally)
library(Hmisc)
library(readxl)
library(cowplot)
library(pwr)
library(ggpubr)
library(psych)
library(lattice)
library(mbir)
library(infer)
library(cvequality)
library(miscor)
library(lmerTest)
library(rmcorr)
library(ICC.Sample.Size)

#power calculations at 0.8 beta and ICC of 0.9 with 3 measures each day
calculateIccSampleSize(p=0.90, p0=0.60, k=3, alpha=0.05, tails=2, power=0.80)

#import data
Subj_Data <- read_excel("~/Desktop/PhD ECU [ACU]/OW UFC Intraday Reliability/Subjective Data Clean.xlsx", sheet = "Form Responses 1")
Delta_Subj <- read_excel("~/Desktop/PhD ECU [ACU]/OW UFC Intraday Reliability/Subjective Data
```

```
Clean.xlsx", sheet = "Delta scores")
View(Subj_Data)
View(Delta_Subj)

OW_Data <- read_excel("~/Desktop/PhD ECU [ACU]/OW UFC Intraday Reliability/OW Raw Data Clean.xlsx", sheet = "OW_data")
Delta_OW <- read_excel("~/Desktop/PhD ECU [ACU]/OW UFC Intraday Reliability/OW Raw Data Clean.xlsx", sheet = "OW_delta")
Loc_OW <- read_excel("~/Desktop/PhD ECU [ACU]/OW UFC Intraday Reliability/OW Raw Data Clean.xlsx", sheet = "OW_diffLoc")
View(OW_Data)
View(Delta_OW)
View(Loc_OW)

#############DESCRIPTIVE DATA AND DIFFERENCES BETWEEN DAYS############
#apply multi.sapply function for descriptive stats
multi.sapply <- function(...) {
  arglist <- match.call(expand.dots = FALSE)$...
  var.names <- sapply(arglist, deparse)
  has.name <- (names(arglist) != "")
  var.names[has.name] <- names(arglist)[has.name]
  arglist <- lapply(arglist, eval.parent, n = 2)
  x <- arglist[[1]]
  arglist[[1]] <- NULL
  result <- sapply(arglist, function (FUN, x) sapply(x, FUN, na.rm=T), x)
  colnames(result) <- var.names[-1]
  return(result)
}

#get overall mean/sd
summary_Subj <- data.frame(multi.sapply(Subj_Data, mean, sd)) %>% rownames_to_column()
summary_OW <- data.frame(multi.sapply(OW_Data, mean, sd)) %>% rownames_to_column()
summary_Subj
summary_OW

#get mean/sd for rest day
Subj_restDay <- Subj_Data %>%
  subset(day_class == 0)

OW_restDay <- OW_Data %>%
  subset(day_class == 0)

Subj_restDay %>%
```

```
  multi.sapply(mean, sd)

OW_restDay %>%
  multi.sapply(mean, sd)

#get mean/sd for moderate day
Subj_modDay <- Subj_Data %>%
  subset(day_class == 1)

OW_modDay <- OW_Data %>%
  subset(day_class == 1)

Subj_modDay %>%
  multi.sapply(mean, sd)

OW_modDay %>%
  multi.sapply(mean, sd)

#get mean/sd for high day
Subj_highDay <- Subj_Data %>%
  subset(day_class == 2)

OW_highDay <- OW_Data %>%
  subset(day_class == 2)

Subj_highDay %>%
  multi.sapply(mean, sd)

OW_highDay %>%
  multi.sapply(mean, sd)

#are there differences between rest day and mod day?
subjDiff_01 <- mapply(function(x, y) smd_test(x, y, paired = F, conf.int = 0.95, swc = 0.2), Subj_restDay[, c("water_prior", "total_trimp", "ppc", "mpc", "eb", "or", "ms", "la", "nes", "or")], Subj_modDay[, c("water_prior", "total_trimp", "ppc", "mpc", "eb", "or", "ms", "la", "nes", "or")])
OWDiff_01 <- mapply(function(x, y) smd_test(x, y, paired = F, conf.int = 0.95, swc = 0.2), OW_restDay[, c("aperiodic_inf", "aspirate_waves", "dc_pot", "hf", "lf", "lf_hf", "mri", "pns", "rmssd", "sdnn", "sns", "tension_index", "total_power", "overal_read", "cardiac_read", "cns_read", "end_read1", "skill_read1", "speedPower_read1", "strength_read1")], OW_modDay[, c("aperiodic_inf", "aspirate_waves", "dc_pot", "hf", "lf", "lf_hf", "mri", "pns", "rmssd", "sdnn", "sns", "tension_index", "total_power", "overal_read", "cardiac_read", "cns_read", "end_read1", "skill_read1", "speedPower_read1", "strength_read1")])
subjDiff_01
OWDiff_01
```

```
#are there differences between rest day and hard day?
subjDiff_02 <- mapply(function(x, y) smd_test(x, y, paired = F, conf.int = 0.95, swc = 0.2), Subj_restDay[, c("water_prior", "total_trimp", "ppc", "mpc", "eb", "or", "ms", "la", "nes", "or")], Subj_highDay[, c("water_prior", "total_trimp", "ppc", "mpc", "eb", "or", "ms", "la", "nes", "or")])
OWDiff_02 <- mapply(function(x, y) smd_test(x, y, paired = F, conf.int = 0.95, swc = 0.2), OW_restDay[, c("aperiodic_inf", "aspirate_waves", "dc_pot", "hf", "lf", "lf_hf", "mri", "pns", "rmssd", "sdnn", "sns", "tension_index", "total_power", "overal_read", "cardiac_read", "cns_read", "end_read1", "skill_read1", "speedPower_read1", "strength_read1")], OW_highDay[, c("aperiodic_inf", "aspirate_waves", "dc_pot", "hf", "lf", "lf_hf", "mri", "pns", "rmssd", "sdnn", "sns", "tension_index", "total_power", "overal_read", "cardiac_read", "cns_read", "end_read1", "skill_read1", "speedPower_read1", "strength_read1")])
subjDiff_02
OWDiff_02

#are there differences between mod day and hard day?
subjDiff_12 <- mapply(function(x, y) smd_test(x, y, paired = F, conf.int = 0.95, swc = 0.2), Subj_modDay[, c("water_prior", "total_trimp", "ppc", "mpc", "eb", "or", "ms", "la", "nes", "or")], Subj_highDay[, c("water_prior", "total_trimp", "ppc", "mpc", "eb", "or", "ms", "la", "nes", "or")])
OWDiff_12 <- mapply(function(x, y) smd_test(x, y, paired = F, conf.int = 0.95, swc = 0.2), OW_modDay[, c("aperiodic_inf", "aspirate_waves", "dc_pot", "hf", "lf", "lf_hf", "mri", "pns", "rmssd", "sdnn", "sns", "tension_index", "total_power", "overal_read", "cardiac_read", "cns_read", "end_read1", "skill_read1", "speedPower_read1", "strength_read1")], OW_highDay[, c("aperiodic_inf", "aspirate_waves", "dc_pot", "hf", "lf", "lf_hf", "mri", "pns", "rmssd", "sdnn", "sns", "tension_index", "total_power", "overal_read", "cardiac_read", "cns_read", "end_read1", "skill_read1", "speedPower_read1", "strength_read1")])
subjDiff_12
OWDiff_12

#convert mean diff r to g (refer back to results of differences between rest, mod and hard days)
r_gConvert01 <- es_convert(c(-0.89, -0.04, -0.06, -0.12, 0.07, 0.04, -0.01, -0.05, 0.04, 0.12, 0.11, -0.19, -0.17, -0.02, -0.16, 0.25, -0.01, 0.09, 0.04, -0.03), from="r", to="d")
r_gConvert02 <- es_convert(c(-0.89, -0.22, 0.29, -0.13, 0.06, 0.17, -0.04, 0.05, 0.11, -0.08, 0.1, 0.06, -0.14, 0.04, 0.07, 0.11, 0.06, -0.17, 0.01, -0.1, -0.05), from="r", to="d")
r_gConvert12 <- es_convert(c(-0.01, 0.12, -0.56, -0.11, 0.05, 0.16, -0.02, -0.01, 0, -0.01, 0, 0.12, 0.05, -0.22, -0.18, -0.08, -0.14, 0.09), from="r", to="d")

###########CORRELATION ANALYSIS#################
#organise data
Delta_Subj <- Delta_Subj[,-c(23:54)]
Delta_OWData <- left_join(Delta_OW, Delta_Subj[,c("initials", "day", "delta_trimp")], by=c("initials", "day"))
Delta_OWDataDC <- Delta_OWData[,c("athlete", "day_class", "delta_trimp")]

#get repeated measures correlations with delta ARSS variables and previous day TRIMP
ppc_trimp_rmcorr <- rmcorr(athlete, delta_trimp, delta_ppc, Delta_Subj)
```

```
mpc_trimp_rmcorr <- rmcorr(athlete, delta_trimp, delta_mpc, Delta_Subj)
eb_trimp_rmcorr <- rmcorr(athlete, delta_trimp, delta_eb, Delta_Subj)
or_trimp_rmcorr <- rmcorr(athlete, delta_trimp, delta_or, Delta_Subj)
ms_trimp_rmcorr <- rmcorr(athlete, delta_trimp, delta_ms, Delta_Subj)
la_trimp_rmcorr <- rmcorr(athlete, delta_trimp, delta_la, Delta_Subj)
nes_trimp_rmcorr <- rmcorr(athlete, delta_trimp, delta_nes, Delta_Subj)
os_trimp_rmcorr <- rmcorr(athlete, delta_trimp, delta_os, Delta_Subj)

#get repeated measures correlations with delta OW variables and previous day TRIMP
dcpot_trimp_rmcorr <- rmcorr(athlete, delta_trimp, delta_dc_pot, Delta_OWData)
ai_trimp_rmcorr <- rmcorr(athlete, delta_trimp, delta_aperiodic, Delta_OWData)
aw_trimp_rmcorr <- rmcorr(athlete, delta_trimp, delta_aspirate_waves, Delta_OWData)
pns_trimp_rmcorr <- rmcorr(athlete, delta_trimp, delta_pns, Delta_OWData)
sns_trimp_rmcorr <- rmcorr(athlete, delta_trimp, delta_sns, Delta_OWData)
ti_trimp_rmcorr <- rmcorr(athlete, delta_trimp, delta_tension_index, Delta_OWData)
hf_trimp_rmcorr <- rmcorr(athlete, delta_trimp, delta_hf, Delta_OWData)
lf_trimp_rmcorr <- rmcorr(athlete, delta_trimp, delta_lf, Delta_OWData)
rmssd_trimp_rmcorr <- rmcorr(athlete, delta_trimp, delta_rmssd, Delta_OWData)
sdnn_trimp_rmcorr <- rmcorr(athlete, delta_trimp, delta_sdnn, Delta_OWData)
sdsd_trimp_rmcorr <- rmcorr(athlete, delta_trimp, delta_sdsd, Delta_OWData)
tp_trimp_rmcorr <- rmcorr(athlete, delta_trimp, delta_total_power, Delta_OWData)

#get repeated measures correlations with OW readiness variables and previous day TRIMP
overallread_trimp_rmcorr <- rmcorr(athlete, delta_trimp, delta_overall_read, Delta_OWData)
cardiacread_trimp_rmcorr <- rmcorr(athlete, delta_trimp, delta_cardiac_read, Delta_OWData)
cnsread_trimp_rmcorr <- rmcorr(athlete, delta_trimp, delta_cns_read, Delta_OWData)
endread_trimp_rmcorr <- rmcorr(athlete, delta_trimp, delta_end_read1, Delta_OWData)
skillread_trimp_rmcorr <- rmcorr(athlete, delta_trimp, delta_skill_read1, Delta_OWData)
speedread_trimp_rmcorr <- rmcorr(athlete, delta_trimp, delta_speedPower_read, Delta_OWData)
strength_trimp_rmcorr <- rmcorr(athlete, delta_trimp, delta_strength_read, Delta_OWData)

#graph corelations on vertical line graph with confidence intervals (forest plot)
#create trimp-ARSS correlation forest plot (check correl data)
trimpARSS_label <- c("PPC", "MPC", "EB", "OR", "MS", "LA", "NES", "OS")
trimpARSS_r    <- c(-0.07, -0.10, -0.01, -0.33, 0.61, 0.41, 0.27, 0.19)
trimpARSS_rLower <- c(-0.47, -0.49, -0.42, -0.65, 0.26, 0.00, -0.16, -0.24)
trimpARSS_rUpper <- c(0.36, 0.33, 0.41, 0.10, 0.82, 0.70, 0.62, 0.56)

trimpARSS_rDf <- data.frame(trimpARSS_label, trimpARSS_r, trimpARSS_rLower,
trimpARSS_rUpper)

# reverses the factor level ordering for labels after coord_flip()
trimpARSS_rDf$trimpARSS_label <- factor(trimpARSS_rDf$trimpARSS_label,
```

```
levels=rev(trimpARSS_rDf$trimpARSS_label))

#title for x axis
r_title <- expression(paste("Previous Day TRIMP ", italic("r"), " [95% CI]"))
x_title1 <- expression(paste(italic("delta"), " ARSS Variables"))
trimpARRSSFor_plot <- ggplot(data=trimpARSS_rDf, aes(x=trimpARSS_label, y=trimpARSS_r, ymin=trimpARSS_rLower, ymax=trimpARSS_rUpper)) +
  geom_pointrange() +
  geom_hline(yintercept=0, lty=2) + # add a dotted line at x=0 after flip
  coord_flip() +  # flip coordinates (puts labels on y axis)
  xlab(x_title1) +
  ylab(r_title) +
  scale_y_continuous(breaks=c(-0.7, -0.5, -0.3, -0.1, 0.1, 0.3, 0.5, 0.7), limits = c(-1, 1)) +
  theme_gray()

print(trimpARRSSFor_plot)

#create trimp-OW correlation forest plot
trimpOW_label <- c("DC", "AI", "AW", "PNS", "SNS", "TI", "HF", "LF", "RMSSD", "SDNN", "SDSD", "TP")
trimpOW_r  <- c(0.16, 0.06, -0.22, -0.13, -0.01, -0.07, 0.02, -0.36, -0.07, 0.06, -0.08, -0.31)
trimpOW_rLower <- c(-0.27, -0.36, -0.58, -0.52, -0.42, -0.47, -0.39, -0.67, -0.47, -0.36, -0.48, -0.64)
trimpOW_rUpper <- c(0.54, 0.46, 0.22, 0.30, 0.40, 0.35, 0.43, 0.06, 0.35, 0.46, 0.34, 0.11)

trimpOW_rDf <- data.frame(trimpOW_label, trimpOW_r, trimpOW_rLower, trimpOW_rUpper)

#reverses the factor level ordering for labels after coord_flip()
trimpOW_rDf$trimpOW_label <- factor(trimpOW_rDf$trimpOW_label, levels=rev(trimpOW_rDf$trimpOW_label))

#title for x axis
x_title2 <- expression(paste(italic("delta"), " Omegawave Raw Variables"))
trimpOWFor_plot <- ggplot(data=trimpOW_rDf, aes(x=trimpOW_label, y=trimpOW_r, ymin=trimpOW_rLower, ymax=trimpOW_rUpper)) +
  geom_pointrange() +
  geom_hline(yintercept=0, lty=2) + # add a dotted line at x=0 after flip
  coord_flip() +  # flip coordinates (puts labels on y axis)
  xlab(x_title2) +
  ylab(r_title) +
  scale_y_continuous(breaks=c(-0.7, -0.5, -0.3, -0.1, 0.1, 0.3, 0.5, 0.7), limits = c(-1, 1)) +
  theme_gray()

print(trimpOWFor_plot)
```

```
#create trimp-OW readiness correlation forest plot
trimpOWread_label <- c("Overall", "Cardiac", "CNS", "Endurance", "Skill", "Speed/Power", "Strength")
trimpOWread_r   <- c(0.18, 0.20, 0.12, 0.31, 0.13, 0.25, 0.25)
trimpOWread_rLower <- c(-0.25, -0.23, -0.31, -0.12, -0.30, -0.19, -0.18)
trimpOWread_rUpper <- c(0.55, 0.56, 0.51, 0.64, 0.51, 0.60, 0.60)

trimpOWread_rDf <- data.frame(trimpOWread_label, trimpOWread_r, trimpOWread_rLower, trimpOWread_rUpper)

#reverses the factor level ordering for labels after coord_flip()
trimpOWread_rDf$trimpOWread_label <- factor(trimpOWread_rDf$trimpOWread_label, levels=rev(trimpOWread_rDf$trimpOWread_label))

#title for x axis
x_title2 <- expression(paste(italic("delta"), " Omegawave Readiness & Windows of Trainability Variables"))
trimpOWreadFor_plot <- ggplot(data=trimpOWread_rDf, aes(x=trimpOWread_label, y=trimpOWread_r, ymin=trimpOWread_rLower, ymax=trimpOWread_rUpper)) +
  geom_pointrange() +
  geom_hline(yintercept=0, lty=2) + # add a dotted line at x=0 after flip
  coord_flip() +   # flip coordinates (puts labels on y axis)
  xlab(x_title2) +
  ylab(r_title) +
  scale_y_continuous(breaks=c(-0.7, -0.5, -0.3, -0.1, 0.1, 0.3, 0.5, 0.7), limits = c(-1, 1)) +
  theme_gray()

print(trimpOWreadFor_plot)

#put all polts together
plot_grid(trimpARRSSFor_plot, trimpOWFor_plot, trimpOWreadFor_plot, label_size = 10, nrow=1)

##########RELIABILITY SECTION######################
#######VARIANCES############
#first check variances between males and females in OW data
#plot data
#organise data
OW_Data1 <- OW_Data[,c("athlete", "sex", "day", "day_class", "measure", "aperiodic_inf", "aspirate_waves", "dc_pot", "dc_potpos", "hf", "lf", "lf_hf", "mri", "pns", "rmssd", "sdnn", "sdsd", "sns", "tension_index", "total_power", "overal_read", "cardiac_read", "cns_read", "end_read1", "skill_read1", "speedPower_read1", "strength_read1")]
logOW_Data <- OW_Data1[,c("athlete", "sex", "day", "day_class", "measure", "aperiodic_inf", "aspirate_waves", "dc_potpos", "hf", "lf", "lf_hf", "mri", "pns", "rmssd", "sdnn", "sdsd", "sns", "tension_index", "total_power")]
```

```
scaleOW_Data <- OW_Data[,c("athlete", "sex", "day", "day_class", "measure", "dc_pot", "overal_read", "cardiac_read", "cns_read", "end_read1", "skill_read1", "speedPower_read1", "strength_read1")]

#plots for variances
ai_fig <- ggplot(OW_Data1, aes(day, aperiodic_inf, color=sex)) + geom_jitter()
asp_fig <- ggplot(OW_Data1, aes(day, aspirate_waves, color=sex)) + geom_jitter()
dc_fig <- ggplot(OW_Data1, aes(day, dc_potpos, color=sex)) + geom_jitter()
hf_fig <- ggplot(OW_Data1, aes(day, hf, color=sex)) + geom_jitter()
lf_fig <- ggplot(OW_Data1, aes(day, lf, color=sex)) + geom_jitter()
lfhf_fig <- ggplot(OW_Data1, aes(day, lf_hf, color=sex)) + geom_jitter()
mri_fig <- ggplot(OW_Data1, aes(day, mri, color=sex)) + geom_jitter()
pns_fig <- ggplot(OW_Data1, aes(day, pns, color=sex)) + geom_jitter()
#to add rmssd, sdsd, sdnn
sns_fig <- ggplot(OW_Data1, aes(day, sns, color=sex)) + geom_jitter()
tension_fig <- ggplot(OW_Data1, aes(day, tension_index, color=sex)) + geom_jitter()
totalpow_fig <- ggplot(OW_Data1, aes(day, total_power, color=sex)) + geom_jitter()
overal_fig <- ggplot(OW_Data1, aes(day, overal_read, color=sex)) + geom_jitter()
cardiac_fig <- ggplot(OW_Data1, aes(day, cardiac_read, color=sex)) + geom_jitter()
cns_fig <- ggplot(OW_Data1, aes(day, cns_read, color=sex)) + geom_jitter()
end_fig <- ggplot(OW_Data1, aes(day, end_read1, color=sex)) + geom_jitter()
skill_fig <- ggplot(OW_Data1, aes(day, skill_read1, color=sex)) + geom_jitter()
speed_fig <- ggplot(OW_Data1, aes(day, speedPower_read1, color=sex)) + geom_jitter()
strength_fig <- ggplot(OW_Data1, aes(day, strength_read1, color=sex)) + geom_jitter()

OW_DataF <- OW_Data1 %>% subset(sex=="f")
OW_DataM <- OW_Data1 %>% subset(sex=="m")

varOW_Data <- data.frame(mapply(function(x, y) var.test(x, y, na.rm=T)$p.value, OW_DataF[,c("aperiodic_inf", "aspirate_waves", "dc_pot", "dc_potpos", "hf", "lf", "lf_hf", "mri", "pns", "sns", "tension_index", "total_power", "overal_read", "cardiac_read", "cns_read", "end_read1", "skill_read1", "speedPower_read1", "strength_read1")], OW_DataM[,c("aperiodic_inf", "aspirate_waves", "dc_potpos", "hf", "lf", "lf_hf", "mri", "pns", "sns", "tension_index", "total_power", "overal_read", "cardiac_read", "cns_read", "end_read1", "skill_read1", "speedPower_read1", "strength_read1")])) %>% rownames_to_column() %>% set_names(c("variable", "Ftest_pvalue"))
varOW_Data %>% filter(Ftest_pvalue < 0.05)

##########FUNCTIONS FOR RELIABILITY##########
#create 100*log function (as per Hopkins)
log100 <- function(x) {100*log(x)}

#create typical error function (as per Hopkins)
te <- function(x, y) {sd(x-y)/sqrt(2)}
#create typical error CI (as per Hopkins)
```

```
#x Deg Freedom, y te
upper95CIFun_chi <- function(x, y) {sqrt(x*(y^2)/qchisq(0.025, x))}
lower95CIFun_chi <- function(x, y) {sqrt(x*(y^2)/qchisq(0.975, x))}
upper95CIFun_norm <- function(x, y) {sqrt(x*(y^2)/qnorm(0.025, x))}
lower95CIFun_norm <- function(x, y) {sqrt(x*(y^2)/qnorm(0.975, x))}

#create ICC CI (as per Hopkins)
F_lower <- function(x, y) {qf(0.025, x, y, lower.tail = F)}
F_upper <- function(x, y) {qf(0.975, x, y, lower.tail = F)}
#x, y Deg Freedom of trials

#create weighted sd function
wtd.sd <- function(x) {sqrt(wtd.var(x))}

#create cv function (if needed for raw variables)
cv <- function(x, y) {(x/y)*100}

###########RELIABILITY BETWEEN DIFFERENT LOCATIONS###########
#get reliability for different locations
#subset groups for at home and at training measures
Loc_logOW <- Loc_OW[,c("athlete", "sex", "day", "day_class", "measure", "aperiodic_inf", "aspirate_waves", "dc_potpos", "hf", "lf", "lf_hf", "mri", "pns", "rmssd", "sdnn", "sdsd", "sns", "tension_index", "total_power")]
Loc_scaleOW <- Loc_OW[,c("athlete", "sex", "day", "day_class", "measure", "dc_pot", "overal_read", "cardiac_read", "cns_read", "end_read1", "skill_read1", "speedPower_read1", "strength_read1")]
Loc1_logOW <- Loc_logOW[,-c(2:4)] %>% subset(measure == 1)
Loc2_logOW <- Loc_logOW[,-c(2:4)] %>% subset(measure == 2)
Loc1_scaleOW <- Loc_scaleOW[,-c(2:4)] %>% subset(measure == 1)
Loc2_scaleOW <- Loc_scaleOW[,-c(2:4)] %>% subset(measure == 2)

#count for measures/deg freedom
Loc1_logOW %>% ungroup() %>% count()
Loc2_logOW %>% ungroup() %>% count()
Loc_n1 <- 20
Loc_n2 <- 20
Loc1_scaleOW %>% ungroup() %>% count()
Loc2_scaleOW %>% ungroup() %>% count()

#LOG VARIABLES
#log typical error between measures at home and at training for log measures
Loc1_logOW <- Loc1_logOW[,-c(1:2)]
Loc2_logOW <- Loc2_logOW[,-c(1:2)]
```

```
te_locLogOW <- data.frame(mapply(function(x, y) te(log100(x), log100(y)), Loc1_logOW, Loc2_logOW))
%>%   rownames_to_column() %>% set_names(c("variable", "log100_te"))
te_locLogOW

#get 95% confidence intervals for typical error of log measures
low95CI_locLogEq <- data.frame(lower95CIFun_chi(19, te_locLogOW$log100_te))
upper95CI_locLogEq <- data.frame(upper95CIFun_chi(19, te_locLogOW$log100_te))

#combine with typical error
teCI_locLogOW <- bind_cols(te_locLogOW, low95CI_locLogEq, upper95CI_locLogEq) %>%
set_names(c("variable", "te", "te_lowCI", "te_upCI"))

#weighted sd for both location log measures
Loc_logOW1 <- Loc_OW[,c("aperiodic_inf", "aspirate_waves", "dc_potpos", "hf", "lf", "lf_hf", "mri", "pns",
"rmssd", "sdnn", "sdsd", "sns", "tension_index", "total_power")]
locLogOWsd <- data.frame(mapply(function(x) wtd.sd(log100(x)), Loc_logOW1)) %>%
rownames_to_column() %>% set_names(c("variable", "overall_sd"))
locLogOWsd

#F values for log variables
locF_lower <- F_lower(19, 19)
LocF_upper <- F_upper(19, 19)

#create Loc reliability table with cv for log variables NOTE: will get NaNs in standardised TE if ICC<0
#first specify n_trials for location measures
n_trialsLoc <- 2
locLogOW_table <- bind_cols(teCI_locLogOW, locLogOWsd) %>%
  mutate(cv_loc = 100*exp(te/100)-100,
         cv_lowCI = 100*exp(te_lowCI/100)-100,
         cv_upCI = 100*exp(te_upCI/100)-100,
         icc_loc = (1-te^2/overall_sd^2)*(1+(1-(1-te^2/overall_sd^2)^2)/(Loc_n1+Loc_n2-(Loc_n2-1)-3)),
         F_value = 1+icc_loc*n_trialsLoc/(1-icc_loc),
         icc_lowCI = ((F_value/locF_lower)-1)/((F_value/locF_lower)+(n_trialsLoc-1)),
         icc_highCI = ((F_value/LocF_upper)-1)/((F_value/LocF_upper)+(n_trialsLoc-1)),
         stand_te = te/sqrt(overall_sd^2-te^2),
         stand_telowCI = te_lowCI/(sqrt(overall_sd^2-te^2)),
         stand_teupCI = te_upCI/(sqrt(overall_sd^2-te^2)))

locLogOW_table1 <- locLogOW_table[,c("variable", "cv_loc", "cv_lowCI", "cv_upCI", "icc_loc",
"icc_lowCI", "icc_highCI", "stand_te", "stand_telowCI", "stand_teupCI")]
View(locLogOW_table1)

#SCALE MEASURES
```

```
#typical error between measures at home and at training for scale measures
Loc1_scaleOW <- Loc1_scaleOW[,-c(1:2)]
Loc2_scaleOW <- Loc2_scaleOW[,-c(1:2)]
te_locScaleOW <- data.frame(mapply(function(x, y) te(x, y), Loc1_scaleOW, Loc2_scaleOW)) %>%
rownames_to_column() %>% set_names(c("variable", "te"))
te_locScaleOW

#get 95% confidence intervals for typical error of log measures
low95CI_locScale <- data.frame(lower95CIFun_chi(19, te_locScaleOW$te))
upper95CI_locScale <- data.frame(upper95CIFun_chi(19, te_locScaleOW$te))

#combine with typical error
teCI_locScaleOW <- bind_cols(te_locScaleOW, low95CI_locScale, upper95CI_locScale) %>%
set_names(c("variable", "te", "te_lowCI", "te_upCI"))

#weighted sd for both location scale measures
Loc_scaleOW1 <- Loc_OW[,c("dc_pot", "overal_read", "cardiac_read", "cns_read", "end_read1",
"skill_read1", "speedPower_read1", "strength_read1")]
locScaleOWsd <- data.frame(mapply(function(x) wtd.sd(x), Loc_scaleOW1)) %>%
rownames_to_column() %>% set_names(c("variable", "overall_sd"))
locScaleOWsd

##create Loc reliability table with cv for log variables NOTE: will get NaNs in standardised TE if ICC<0
locF_lower <- F_lower(19, 19)
LocF_upper <- F_upper(19, 19)

#create Loc reliability table for scale variables
locScaleOW_table <- bind_cols(teCI_locScaleOW, locScaleOWsd) %>%
  mutate(icc_loc = (1-te^2/overall_sd^2)*(1+(1-(1-te^2/overall_sd^2)^2)/(Loc_n1+Loc_n2-(Loc_n2-1)-3)),
         F_value = 1+icc_loc*n_trialsLoc/(1-icc_loc),
         icc_lowCI = ((F_value/locF_lower)-1)/((F_value/locF_lower)+(n_trialsLoc-1)),
         icc_highCI = ((F_value/LocF_upper)-1)/((F_value/LocF_upper)+(n_trialsLoc-1)),
         stand_te = te/sqrt(overall_sd^2-te^2),
         stand_telowCI = te_lowCI/(sqrt(overall_sd^2-te^2)),
         stand_teupCI = te_upCI/(sqrt(overall_sd^2-te^2))) %>%
  select(-c("variable1", "overall_sd", "F_value"))

locScaleOW_table

####################################3

########INTRADAY RELIABILITY DATA#############
#LOG MEASURES
```

```
#filter groups for repeated training measures for rest day
logOWt1_rest <- logOW_Data %>% filter(measure == 2, day_class == 0)
logOWt2_rest <- logOW_Data %>% filter(measure == 3, day_class == 0)
logOWt3_rest <- logOW_Data %>% filter(measure == 4, day_class == 0)

#filter groups for repeated training measures for moderate day
logOWt1_rest <- logOWt1_rest[,-c(1:5)]
logOWt2_rest <- logOWt2_rest[,-c(1:5)]
logOWt3_rest <- logOWt3_rest[,-c(1:5)]

logOWt12_rest <- bind_rows(logOWt1_rest, logOWt2_rest)
logOWt23_rest <- bind_rows(logOWt2_rest, logOWt3_rest)
logOWt123_rest <- bind_rows(logOWt1_rest, logOWt2_rest, logOWt3_rest)

#filter groups for repeated training measures for moderate day
logOWt1_mod <- logOW_Data %>% group_by(athlete) %>% filter(measure == 2, day_class == 1)
logOWt2_mod <- logOW_Data %>% group_by(athlete) %>% filter(measure == 3, day_class == 1)
logOWt3_mod <- logOW_Data %>% group_by(athlete) %>% filter(measure == 4, day_class == 1)

logOWt1_mod <- logOWt1_mod[,-c(2:5)]
logOWt2_mod1 <- logOWt2_mod[,-c(2:5)]
logOWt2_mod2 <- logOWt2_mod[,-c(2:5)] %>% filter(athlete != 3)
#athlete 3 didnt complete 3rd test on this day
logOWt3_mod <- logOWt3_mod[,-c(2:5)]

logOWt12_mod <- bind_rows(logOWt1_mod, logOWt2_mod)
logOWt23_mod <- bind_rows(logOWt2_mod, logOWt3_mod) %>% filter(athlete != 3)
#athlete 3 didnt complete 3rd test on this day
logOWt123_mod <- bind_rows(logOWt1_mod, logOWt2_mod, logOWt3_mod)

#filter groups for repeated training measures for hard day
logOWt1_hard <- logOW_Data %>% filter(measure == 2, day_class == 2)
logOWt2_hard <- logOW_Data %>% filter(measure == 3, day_class == 2)
logOWt3_hard <- logOW_Data %>% filter(measure == 4, day_class == 2)

logOWt1_hard <- logOWt1_hard[,-c(1:5)]
logOWt2_hard <- logOWt2_hard[,-c(1:5)]
logOWt3_hard <- logOWt3_hard[,-c(1:5)]

logOWt12_hard <- bind_rows(logOWt1_hard, logOWt2_hard)
logOWt23_hard <- bind_rows(logOWt2_hard, logOWt3_hard)
logOWt123_hard <- bind_rows(logOWt1_hard, logOWt2_hard, logOWt3_hard)
```

```
#count for measures
n_trialsIntra <- 3
logOWt1_rest %>% ungroup() %>% count()
logOWt2_rest %>% ungroup() %>% count()
logOWt3_rest %>% ungroup() %>% count()
logOWRest_n <- 12
df_rest <- logOWRest_n-1

logOWt1_mod %>% ungroup() %>% count()
logOWt2_mod %>% ungroup() %>% count()
logOWt3_mod %>% ungroup() %>% count()
logOWMod_n <- 13
df_mod <- logOWMod_n-1

logOWt1_hard %>% ungroup() %>% count()
logOWt2_hard %>% ungroup() %>% count()
logOWt3_hard %>% ungroup() %>% count()
logOWHard_n <- 13
df_hard <- logOWHard_n-1

#create rest day reliability table
#typical error between measures t1-2 on rest day
te_t12RestlogOW <- data.frame(mapply(function(x, y) te(log100(x), log100(y)), logOWt2_rest, logOWt1_rest)) %>% rownames_to_column() %>% set_names(c("variable", "te_t12Rest"))
te_t12RestlogOW

#typical error between measures t2-3 on rest day
te_t23RestlogOW <- data.frame(mapply(function(x, y) te(log100(x), log100(y)), logOWt3_rest, logOWt2_rest)) %>% rownames_to_column() %>% set_names(c("variable", "te_t23Rest"))
te_t23RestlogOW

#combine t12 and t23
te_RestlogOW <- bind_cols(te_t12RestlogOW, te_t23RestlogOW) %>%
  as.data.frame() %>% rowwise() %>%
  mutate(te_Rest = sqrt(((te_t12Rest*te_t12Rest*df_rest)+(te_t23Rest*te_t23Rest*df_rest))/(df_rest*(n_trialsIntra-1))))

#get 95% CI for te
df_Restmean <- (1-0.22*(logOWRest_n*n_trialsIntra)/(n_trialsIntra*logOWRest_n))*((logOWRest_n-1)*(n_trialsIntra-1))
low95CI_LogRest <- data.frame(lower95CIFun_chi(df_Restmean, te_RestlogOW$te_Rest))
up95CI_LogRest <- data.frame(upper95CIFun_chi(df_Restmean, te_RestlogOW$te_Rest))
```

```
#combine with typical error
teCI_LogRest <- bind_cols(te_RestlogOW, low95CI_LogRest, up95CI_LogRest) %>% select(-c(2:4)) %>%
  set_names(c("variable", "te", "te_lowCI", "te_upCI"))

#weighted sd for Rest log measures
RestLogOWsd <- data.frame(mapply(function(x) wtd.sd(log100(x)), logOWt123_rest)) %>%
rownames_to_column() %>% set_names(c("variable", "overall_sd"))
RestLogOWsd

#F values for log variables
logF_lowerRest <- F_lower(logOWRest_n-1, df_Restmean)
logF_upperRest <- F_upper(logOWRest_n-1, df_Restmean)

#create Rest reliability table with cv for Log variables
RestLogOW_table <- bind_cols(teCI_LogRest, RestLogOWsd) %>%
  select(-5) %>%
  mutate(cv_rest = 100*exp(te/100)-100,
         cv_lowCI = 100*exp(te_lowCI/100)-100,
         cv_upCI = 100*exp(te_upCI/100)-100,
         icc_rest = (1-te^2/overall_sd^2)*(1+(1-(1-te^2/overall_sd^2)^2)/(logOWRest_n-3)),
         F_value = 1+icc_rest*(1+df_Restmean/(logOWRest_n-1))/(1-icc_rest),
         icc_lowCI = ((F_value/logF_lowerRest)-
1)/((F_value/logF_lowerRest)+((1+df_Restmean/(logOWRest_n-1)-1))),
         icc_highCI = ((F_value/logF_upperRest)-
1)/((F_value/logF_upperRest)+((1+df_Restmean/(logOWRest_n-1)-1))),
         stand_te = te/sqrt(overall_sd^2),
         stand_telowCI = te_lowCI/(sqrt(overall_sd^2)),
         stand_teupCI = te_upCI/(sqrt(overall_sd^2))) %>%
  select("variable", "te", "cv_rest", "icc_rest")

RestLogOW_table

#SCALE VARIABLES
#filter groups for repeated training measures for rest day
scaleOWt1_rest <- scaleOW_Data %>% filter(measure == 2, day_class == 0)
scaleOWt2_rest <- scaleOW_Data %>% filter(measure == 3, day_class == 0)
scaleOWt3_rest <- scaleOW_Data %>% filter(measure == 4, day_class == 0)

#filter groups for repeated training measures for moderate day
scaleOWt1_rest <- scaleOWt1_rest[,-c(1:5)]
scaleOWt2_rest <- scaleOWt2_rest[,-c(1:5)]
scaleOWt3_rest <- scaleOWt3_rest[,-c(1:5)]
```

```
scaleOWt12_rest <- bind_rows(scaleOWt1_rest, scaleOWt2_rest)
scaleOWt23_rest <- bind_rows(scaleOWt2_rest, scaleOWt3_rest)
scaleOWt123_rest <- bind_rows(scaleOWt1_rest, scaleOWt2_rest, scaleOWt3_rest)

#filter groups for repeated training measures for moderate day
scaleOWt1_mod <- scaleOW_Data %>% group_by(athlete) %>% filter(measure == 2, day_class == 1)
scaleOWt2_mod <- scaleOW_Data %>% group_by(athlete) %>% filter(measure == 3, day_class == 1)
scaleOWt3_mod <- scaleOW_Data %>% group_by(athlete) %>% filter(measure == 4, day_class == 1)

scaleOWt1_mod <- scaleOWt1_mod[,-c(2:5)]
scaleOWt2_mod1 <- scaleOWt2_mod[,-c(2:5)]
scaleOWt2_mod2 <- scaleOWt2_mod[,-c(2:5)] %>% filter(athlete != 3)
#athlete 3 didnt complete 3rd test on this day
scaleOWt3_mod <- scaleOWt3_mod[,-c(2:5)]

scaleOWt12_mod <- bind_rows(scaleOWt1_mod, scaleOWt1_mod)
scaleOWt23_mod <- bind_rows(scaleOWt2_mod, scaleOWt3_mod) %>% filter(athlete != 3)
#athlete 3 didnt complete 3rd test on this day
scaleOWt123_mod <- bind_rows(scaleOWt1_mod, scaleOWt2_mod, scaleOWt3_mod)

#filter groups for repeated training measures for hard day
scaleOWt1_hard <- scaleOW_Data %>% filter(measure == 2, day_class == 2)
scaleOWt2_hard <- scaleOW_Data %>% filter(measure == 3, day_class == 2)
scaleOWt3_hard <- scaleOW_Data %>% filter(measure == 4, day_class == 2)

scaleOWt1_hard <- scaleOWt1_hard[,-c(1:5)]
scaleOWt2_hard <- scaleOWt2_hard[,-c(1:5)]
scaleOWt3_hard <- scaleOWt3_hard[,-c(1:5)]

scaleOWt12_hard <- bind_rows(scaleOWt1_hard, scaleOWt2_hard)
scaleOWt23_hard <- bind_rows(scaleOWt2_hard, scaleOWt3_hard)
scaleOWt123_hard <- bind_rows(scaleOWt1_hard, scaleOWt2_hard, scaleOWt3_hard)

#count for measures
n_trialsIntra <- 3
scaleOWt1_rest %>% ungroup() %>% count()
scaleOWt2_rest %>% ungroup() %>% count()
scaleOWt3_rest %>% ungroup() %>% count()
scaleOWRest_n <- 12

scaleOWt1_mod %>% ungroup() %>% count()
scaleOWt2_mod %>% ungroup() %>% count()
scaleOWt3_mod %>% ungroup() %>% count()
```

```
scaleOWMod_n <- 13

scaleOWt1_hard %>% ungroup() %>% count()
scaleOWt2_hard %>% ungroup() %>% count()
scaleOWt3_hard %>% ungroup() %>% count()
scaleOWHard_n <- 13

#create rest day reliability table
#typical error between measures t1-2 on rest day
te_t12RestscaleOW <- data.frame(mapply(function(x, y) te(x, y), scaleOWt2_rest, scaleOWt1_rest)) %>%
rownames_to_column() %>% set_names(c("variable", "te_t12Rest"))
te_t12RestscaleOW

#typical error between measures t2-3 on rest day
te_t23RestscaleOW <- data.frame(mapply(function(x, y) te(x, y), scaleOWt3_rest, scaleOWt2_rest)) %>%
rownames_to_column() %>% set_names(c("variable", "te_t23Rest"))
te_t23RestscaleOW

#combine t12 and t23
te_RestscaleOW <- bind_cols(te_t12RestscaleOW, te_t23RestscaleOW) %>% select(-3) %>%
  as.data.frame() %>% rowwise() %>%
  mutate(te_Rest =
sqrt(((te_t12Rest*te_t12Rest*df_rest)+(te_t23Rest*te_t23Rest*df_rest))/(df_rest*(n_trialsIntra-1)))) %>%
  select(-c(2,3))

#get 95% CI for te
low95CI_ScaleRest <- data.frame(lower95CIFun_chi(df_Restmean, te_RestscaleOW$te_Rest))
up95CI_ScaleRest <- data.frame(upper95CIFun_chi(df_Restmean, te_RestscaleOW$te_Rest))

#combine with typical error
teCI_ScaleRest <- bind_cols(te_RestscaleOW, low95CI_ScaleRest, up95CI_ScaleRest) %>%
set_names(c("variable", "te", "te_lowCI", "te_upCI"))

#weighted sd for Rest log measures
RestScaleOWsd <- data.frame(mapply(function(x) wtd.sd(x), scaleOWt123_rest)) %>%
rownames_to_column() %>% set_names(c("variable", "overall_sd"))
RestScaleOWsd

#F values for scale variables
scaleF_lowerRest <- F_lower(scaleOWRest_n-1, df_Restmean)
scaleF_upperRest <- F_upper(scaleOWRest_n-1, df_Restmean)

#create Rest reliability table with cv for Log variables
```

```
RestScaleOW_table <- bind_cols(teCI_ScaleRest, RestScaleOWsd) %>%
  select(-5) %>%
  mutate(icc_rest = (1-te^2/overall_sd^2)*(1+(1-(1-te^2/overall_sd^2)^2)/(scaleOWRest_n-3)),
         F_value = 1+icc_rest*(1+df_Restmean/(scaleOWRest_n-1))/(1-icc_rest),
         icc_lowCI = ((F_value/scaleF_lowerRest)-1)/((F_value/scaleF_lowerRest)+((1+df_Restmean/(scaleOWRest_n-1)-1))),
         icc_highCI = ((F_value/scaleF_upperRest)-1)/((F_value/scaleF_upperRest)+((1+df_Restmean/(scaleOWRest_n-1)-1))),
         stand_te = te/sqrt(overall_sd^2),
         stand_telowCI = te_lowCI/(sqrt(overall_sd^2)),
         stand_teupCI = te_upCI/(sqrt(overall_sd^2))) %>%
  select("variable", "te", "icc_rest")

RestScaleOW_table

##################
#MODERATE DAY
#LOG VARIABLES
#create mod day reliability table
#typical error between measures t1-2 on mod day
te_t12ModlogOW <- data.frame(mapply(function(x, y) te(log100(x), log100(y)), logOWt2_mod1, logOWt1_mod)) %>% rownames_to_column() %>% set_names(c("variable", "te_t12Mod"))
te_t12ModlogOW

#typical error between measures t2-3 on moderate day
#use second measure 2 recordinngs ie. logOWt2_mod2
te_t23ModlogOW <- data.frame(mapply(function(x, y) te(log100(x), log100(y)), logOWt3_mod, logOWt2_mod2)) %>% rownames_to_column() %>% set_names(c("variable", "te_t23Mod"))
te_t23ModlogOW

#combine t12 and t23
te_ModlogOW <- bind_cols(te_t12ModlogOW, te_t23ModlogOW) %>% select(-3) %>%
  as.data.frame() %>% rowwise() %>%
  mutate(te_Mod = sqrt(((te_t12Mod*te_t12Mod*df_mod)+(te_t23Mod*te_t23Mod*df_mod))/(df_mod*(n_trialsIntra-1)))) %>%
  select(-c(2,3))

#get 95% CI for te
df_Modmean <- (1-0.22*(logOWMod_n*n_trialsIntra)/(n_trialsIntra*logOWMod_n))*((logOWMod_n-1)*(n_trialsIntra-1))
low95CI_LogMod <- data.frame(lower95CIFun_chi(df_Modmean, te_ModlogOW$te_Mod))
up95CI_LogMod <- data.frame(upper95CIFun_chi(df_Modmean, te_ModlogOW$te_Mod))
```

```
#combine with typical error
teCI_LogMod <- bind_cols(te_ModlogOW, low95CI_LogMod, up95CI_LogMod) %>%
set_names(c("variable", "te", "te_lowCI", "te_upCI"))

#weighted sd for moderate log measures
ModLogOWsd <- data.frame(mapply(function(x) wtd.sd(log100(x)), logOWt123_mod[,c(1:15)])) %>%
rownames_to_column() %>% set_names(c("variable", "overall_sd"))
ModLogOWsd

#F values for log variables
logF_lowerMod <- F_lower(logOWMod_n-1, df_Modmean)
logF_upperMod <- F_upper(logOWMod_n-1, df_Modmean)

#create moderate reliability table with cv for Log variables
ModLogOW_table <- bind_cols(teCI_LogMod, ModLogOWsd) %>%
  select(-5) %>%
  mutate(cv_mod = 100*exp(te/100)-100,
         cv_lowCI = 100*exp(te_lowCI/100)-100,
         cv_upCI = 100*exp(te_upCI/100)-100,
         icc_mod = (1-te^2/overall_sd^2)*(1+(1-(1-te^2/overall_sd^2)^2)/(logOWMod_n-3)),
         F_value = 1+icc_mod*(1+df_Modmean/(logOWMod_n-1))/(1-icc_mod),
         icc_lowCI = ((F_value/logF_lowerMod)-
1)/((F_value/logF_lowerMod)+((1+df_Modmean/(logOWMod_n-1)-1))),
         icc_highCI = ((F_value/logF_upperMod)-
1)/((F_value/logF_upperMod)+((1+df_Modmean/(logOWMod_n-1)-1))),
         stand_te = te/sqrt(overall_sd^2),
         stand_telowCI = te_lowCI/(sqrt(overall_sd^2)),
         stand_teupCI = te_upCI/(sqrt(overall_sd^2))) %>%
  select("variable", "te", "cv_mod", "icc_mod")

ModLogOW_table

#SCALE VARIABLES
#create mod day reliability table
#typical error between measures t1-2 on moderate day
te_t12ModscaleOW <- data.frame(mapply(function(x, y) te(x, y), scaleOWt2_mod1, scaleOWt1_mod))
%>% rownames_to_column() %>% set_names(c("variable", "te_t12Mod"))
te_t12ModscaleOW

#typical error between measures t2-3 on moderate day
#use second messure 2 recordinngs ie. logOWt2_mod2
te_t23ModscaleOW <- data.frame(mapply(function(x, y) te(x, y), scaleOWt3_mod, scaleOWt2_mod2))
```

```
%>% rownames_to_column() %>% set_names(c("variable", "te_t23Mod"))
te_t23ModscaleOW

#combine t12 and t23
te_ModscaleOW <- bind_cols(te_t12ModscaleOW, te_t23ModscaleOW) %>% select(-3) %>%
  as.data.frame() %>% rowwise() %>%
  mutate(te_Mod =
sqrt(((te_t12Mod*te_t12Mod*df_mod)+(te_t23Mod*te_t23Mod*df_mod))/(df_mod*(n_trialsIntra-1))))
%>%
  select(-c(2,3))

#get 95% CI for te
low95CI_ScaleMod <- data.frame(lower95CIFun_chi(df_Modmean, te_ModscaleOW$te_Mod))
up95CI_ScaleMod <- data.frame(upper95CIFun_chi(df_Modmean, te_ModscaleOW$te_Mod))

#combine with typical error
teCI_ScaleMod <- bind_cols(te_ModscaleOW, low95CI_ScaleMod, up95CI_ScaleMod) %>%
set_names(c("variable", "te", "te_lowCI", "te_upCI"))

#weighted sd for moderate scale measures
ModScaleOWsd <- data.frame(mapply(function(x) wtd.sd(x), scaleOWt123_mod)) %>%
rownames_to_column() %>% set_names(c("variable", "overall_sd"))
ModScaleOWsd <- ModScaleOWsd[c(1:9),]

#F values for scale variables
scaleF_lowerMod <- F_lower(scaleOWMod_n-1, df_Modmean)
scaleF_upperMod <- F_upper(scaleOWMod_n-1, df_Modmean)

#create moderate reliability table for scale variables
ModScaleOW_table <- bind_cols(teCI_ScaleMod, ModScaleOWsd) %>%
  select(-5) %>%
  mutate(icc_mod = (1-te^2/overall_sd^2)*(1+(1-(1-te^2/overall_sd^2)^2)/(logOWMod_n-3)),
         F_value = 1+icc_mod*(1+df_Modmean/(logOWMod_n-1))/(1-icc_mod),
         icc_lowCI = ((F_value/logF_lowerMod)-
1)/((F_value/logF_lowerMod)+((1+df_Modmean/(logOWMod_n-1)-1))),
         icc_highCI = ((F_value/logF_upperMod)-
1)/((F_value/logF_upperMod)+((1+df_Modmean/(logOWMod_n-1)-1))),
         stand_te = te/sqrt(overall_sd^2),
         stand_telowCI = te_lowCI/(sqrt(overall_sd^2)),
         stand_teupCI = te_upCI/(sqrt(overall_sd^2))) %>%
  select("variable", "te", "icc_mod")

ModScaleOW_table
```

```
##################
#HARD DAY
#LOG VARIABLES
#create hard day reliability table
#typical error between measures t1-2 on hard day
te_t12HardlogOW <- data.frame(mapply(function(x, y) te(log100(x), log100(y)), logOWt2_hard, logOWt1_hard)) %>% rownames_to_column() %>% set_names(c("variable", "te_t12Hard"))
te_t12HardlogOW

#typical error between measures t2-3 on hard day
te_t23HardlogOW <- data.frame(mapply(function(x, y) te(log100(x), log100(y)), logOWt3_hard, logOWt2_hard)) %>% rownames_to_column() %>% set_names(c("variable", "te_t23Hard"))
te_t23HardlogOW

#combine t12 and t23
te_HardlogOW <- bind_cols(te_t12HardlogOW, te_t23HardlogOW) %>% select(-3) %>%
  as.data.frame() %>% rowwise() %>%
  mutate(te_Hard =
sqrt(((te_t12Hard*te_t12Hard*df_hard)+(te_t23Hard*te_t23Hard*df_hard))/(df_hard*(n_trialsIntra-1))))
%>%
  select(-c(2,3))

#get 95% CI for te
df_Hardmean <- (1-0.22*(logOWHard_n*n_trialsIntra)/(n_trialsIntra*logOWHard_n))*((logOWHard_n-1)*(n_trialsIntra-1))
low95CI_LogHard <- data.frame(lower95CIFun_chi(df_Hardmean, te_HardlogOW$te_Hard))
up95CI_LogHard <- data.frame(upper95CIFun_chi(df_Hardmean, te_HardlogOW$te_Hard))

#combine with typical error
teCI_LogHard <- bind_cols(te_HardlogOW, low95CI_LogHard, up95CI_LogHard) %>% set_names(c("variable", "te", "te_lowCI", "te_upCI"))

#weighted sd for hard log measures
HardLogOWsd <- data.frame(mapply(function(x) wtd.sd(log100(x)), logOWt123_hard)) %>% rownames_to_column() %>% set_names(c("variable", "overall_sd"))
HardLogOWsd

#F values for log variables
logF_lowerHard <- F_lower(logOWHard_n-1, df_Hardmean)
logF_upperHard <- F_upper(logOWHard_n-1, df_Hardmean)

#create hard reliability table with cv for Log variables
```

```
HardLogOW_table <- bind_cols(teCI_LogHard, HardLogOWsd) %>%
  select(-5) %>%
  mutate(cv_hard = 100*exp(te/100)-100,
         cv_lowCI = 100*exp(te_lowCI/100)-100,
         cv_upCI = 100*exp(te_upCI/100)-100,
         icc_hard = (1-te^2/overall_sd^2)*(1+(1-(1-te^2/overall_sd^2)^2)/(logOWHard_n-3)),
         F_value = 1+icc_hard*(1+df_Hardmean/(logOWHard_n-1))/(1-icc_hard),
         icc_lowCI = ((F_value/logF_lowerHard)-1)/((F_value/logF_lowerHard)+((1+df_Hardmean/(logOWHard_n-1)-1))),
         icc_highCI = ((F_value/logF_upperHard)-1)/((F_value/logF_upperHard)+((1+df_Hardmean/(logOWHard_n-1)-1))),
         stand_te = te/sqrt(overall_sd^2),
         stand_telowCI = te_lowCI/(sqrt(overall_sd^2)),
         stand_teupCI = te_upCI/(sqrt(overall_sd^2))) %>%
  select("variable", "te", "cv_hard", "icc_hard")

HardLogOW_table

#SCALE VARIABLES
#create hard day reliability table
#typical error between measures t1-2 on hard day
te_t12HardscaleOW <- data.frame(mapply(function(x, y) te(x, y), scaleOWt2_hard, scaleOWt1_hard))
%>% rownames_to_column() %>% set_names(c("variable", "te_t12Hard"))
te_t12HardscaleOW

#typical error between measures t2-3 on hard day
te_t23HardscaleOW <- data.frame(mapply(function(x, y) te(x, y), scaleOWt3_hard, scaleOWt2_hard))
%>% rownames_to_column() %>% set_names(c("variable", "te_t23Hard"))
te_t23HardscaleOW

#combine t12 and t23
te_HardscaleOW <- bind_cols(te_t12HardscaleOW, te_t23HardscaleOW) %>% select(-3) %>%
  as.data.frame() %>% rowwise() %>%
  mutate(te_Hard = sqrt(((te_t12Hard*te_t12Hard*df_hard)+(te_t23Hard*te_t23Hard*df_hard))/(df_hard*(n_trialsIntra-1))))
%>%
  select(-c(2,3))

#get 95% CI for te
low95CI_ScaleHard <- data.frame(lower95CIFun_chi(df_Hardmean, te_HardscaleOW$te_Hard))
up95CI_ScaleHard <- data.frame(upper95CIFun_chi(df_Hardmean, te_HardscaleOW$te_Hard))

#combine with typical error
```

```
teCI_ScaleHard <- bind_cols(te_HardscaleOW, low95CI_ScaleHard, up95CI_ScaleHard) %>%
set_names(c("variable", "te", "te_lowCI", "te_upCI"))

#weighted sd for hard scale measures
HardScaleOWsd <- data.frame(mapply(function(x) wtd.sd(x), scaleOWt123_hard)) %>%
rownames_to_column() %>% set_names(c("variable", "overall_sd"))

#F values for scale variables
scaleF_lowerHard <- F_lower(scaleOWHard_n-1, df_Hardmean)
scaleF_upperHard <- F_upper(scaleOWHard_n-1, df_Hardmean)

#create hard reliability table for scale variables
HardScaleOW_table <- bind_cols(teCI_ScaleHard, HardScaleOWsd) %>%
  select(-5) %>%
  mutate(icc_hard = (1-te^2/overall_sd^2)*(1+(1-(1-te^2/overall_sd^2)^2)/(logOWHard_n-3)),
         F_value = 1+icc_hard*(1+df_Hardmean/(logOWHard_n-1))/(1-icc_hard),
         icc_lowCI = ((F_value/scaleF_lowerHard)-
1)/((F_value/scaleF_lowerHard)+((1+df_Hardmean/(logOWHard_n-1)-1))),
         icc_highCI = ((F_value/scaleF_upperHard)-
1)/((F_value/scaleF_upperHard)+((1+df_Hardmean/(logOWHard_n-1)-1))),
         stand_te = te/sqrt(overall_sd^2),
         stand_telowCI = te_lowCI/(sqrt(overall_sd^2)),
         stand_teupCI = te_upCI/(sqrt(overall_sd^2))) %>%
  select("variable", "te", "icc_hard")

HardScaleOW_table

######################
#OVERALL RELIABILITY
#LOG VARIABLES
#get overall mean TE/CV, ICC and standardised TE & icc for all three days
logOWOverall_Rel <- bind_cols(RestLogOW_table, ModLogOW_table[c(2:15),], HardLogOW_table)
%>%
  select("variable", "te", "te1", "te2") %>%
  mutate(te_Overall =
sqrt(((te*te*df_rest)+(te1*te1*df_mod)+(te2*te2*df_hard))/(df_rest+df_mod+df_hard))) %>%
  select(-c(2:4))

#get 95% CI for te
low95CI_LogOverall <- data.frame(lower95CIFun_chi(df_Hardmean, logOWOverall_Rel$te_Overall))
up95CI_LogOverall <- data.frame(upper95CIFun_chi(df_Hardmean, logOWOverall_Rel$te_Overall))

#combine with typical error
```

```
logOWOverall_Rel1 <- bind_cols(logOWOverall_Rel, low95CI_LogOverall, up95CI_LogOverall) %>%
set_names(c("variable", "te", "te_lowCI", "te_upCI"))

#weighted sd for overall log measures
#first combine hard, mod and easy days
logOWt123_overall <- bind_rows(logOWt123_rest, logOWt123_mod[,c(2:15)], logOWt123_hard)
OverallLogOWsd <- data.frame(mapply(function(x) wtd.sd(log100(x)), logOWt123_overall)) %>%
rownames_to_column() %>% set_names(c("variable", "overall_sd"))

#F values for log variables
#keep these same as trials are on seperate days
logF_lowerOverall <- F_lower(logOWHard_n-1, df_Hardmean)
logF_upperOverall <- F_upper(logOWHard_n-1, df_Hardmean)

#create overall reliability table with cv for Log variables
#kept df and n same as trials are on seperate days
OverallLogOW_table <- bind_cols(logOWOverall_Rel1, OverallLogOWsd) %>%
  select(-5) %>%
  mutate(cv_over = 100*exp(te/100)-100,
         cv_lowCI = 100*exp(te_lowCI/100)-100,
         cv_upCI = 100*exp(te_upCI/100)-100,
         icc_over = (1-te^2/overall_sd^2)*(1+(1-(1-te^2/overall_sd^2)^2)/(logOWHard_n-3)),
         F_value = 1+icc_over*(1+df_Hardmean/(logOWHard_n-1))/(1-icc_over),
         icc_lowCI = ((F_value/logF_lowerOverall)-
1)/((F_value/logF_lowerOverall)+((1+df_Hardmean/(logOWHard_n-1)-1))),
         icc_highCI = ((F_value/logF_upperOverall)-
1)/((F_value/logF_upperOverall)+((1+df_Hardmean/(logOWHard_n-1)-1))),
         stand_te = te/sqrt(overall_sd^2),
         stand_telowCI = te_lowCI/(sqrt(overall_sd^2)),
         stand_teupCI = te_upCI/(sqrt(overall_sd^2))) %>%
  select(-c(2:5))

OverallLogOW_table
View(OverallLogOW_table)

#SCALE VARIABLES
#get overall mean TE/CV, ICC and standardised TE & icc for all three days
scaleOWOverall_Rel <- bind_cols(RestScaleOW_table, ModScaleOW_table[c(2:9),], HardScaleOW_table)
%>%
  select(c(variable, te, te1, te2)) %>%
  mutate(te_Overall =
sqrt(((te*te*df_rest)+(te1*te1*df_mod)+(te2*te2*df_hard))/(df_rest+df_mod+df_hard))) %>%
  select(-c(2:4))
```

```
#get 95% CI for te
low95CI_ScaleOverall <- data.frame(lower95CIFun_chi(df_Hardmean, scaleOWOverall_Rel$te_Overall))
up95CI_ScaleOverall <- data.frame(upper95CIFun_chi(df_Hardmean, scaleOWOverall_Rel$te_Overall))

#combine with typical error
scaleOWOverall_Rel1 <- bind_cols(scaleOWOverall_Rel, low95CI_ScaleOverall, up95CI_ScaleOverall) %>% set_names(c("variable", "te", "te_lowCI", "te_upCI"))

#weighted sd for overall scale measures
#first combine hard, mod and easy days
#moderate day has extra due to one less test on 3rd measure
scaleOWt123_overall <- bind_rows(scaleOWt123_rest, scaleOWt123_mod[,c(2:9)], scaleOWt123_hard)
OverallScaleOWsd <- data.frame(mapply(function(x) wtd.sd(x), scaleOWt123_overall)) %>% rownames_to_column() %>% set_names(c("variable", "overall_sd"))

#F values for scale variables
#keep these same as trials are on seperate days
scaleF_lowerOverall <- F_lower(scaleOWHard_n-1, df_Hardmean)
scaleF_upperOverall <- F_upper(scaleOWHard_n-1, df_Hardmean)

#create overall reliability table with cv for scale variables
#kept df and n same as trials are on seperate days
OverallScaleOW_table <- bind_cols(scaleOWOverall_Rel1, OverallScaleOWsd) %>%
  select(-5) %>%
  mutate(icc_over = (1-te^2/overall_sd^2)*(1+(1-(1-te^2/overall_sd^2)^2)/(scaleOWHard_n-3)),
         F_value = 1+icc_over*(1+df_Hardmean/(scaleOWHard_n-1))/(1-icc_over),
         icc_lowCI = ((F_value/scaleF_lowerOverall)-1)/((F_value/scaleF_lowerOverall)+((1+df_Hardmean/(scaleOWHard_n-1)-1))),
         icc_highCI = ((F_value/scaleF_upperOverall)-1)/((F_value/scaleF_upperOverall)+((1+df_Hardmean/(scaleOWHard_n-1)-1))),
         stand_te = te/sqrt(overall_sd^2),
         stand_telowCI = te_lowCI/(sqrt(overall_sd^2)),
         stand_teupCI = te_upCI/(sqrt(overall_sd^2)))

OverallScaleOW_table
View(OverallScaleOW_table)

##################
########INTERDAY CV/TE FOR SIGNAL TO NOISE RATIO#########
#get interday CV from t1 log measures
#no t1 measure on rest day from athlete 12
```

```
#log variables
logOWt1_rest1 <- logOW_Data %>% filter(measure == 2, day_class == 0) %>% filter(athlete != 12) %>%
select(-c(2:5))
logOWt1_mod1 <- logOW_Data %>% filter(measure == 2, day_class == 1) %>% filter(athlete != 12) %>%
select(-c(2:5))
logOWt1_hard1 <- logOW_Data %>% filter(measure == 2, day_class == 2) %>% filter(athlete != 12) %>%
select(-c(2:5))

#scale variables
scaleOWt1_rest1 <- scaleOW_Data %>% filter(measure == 2, day_class == 0) %>% filter(athlete != 12) %>%
select(-c(2:5))
scaleOWt1_mod1 <- scaleOW_Data %>% filter(measure == 2, day_class == 1) %>% filter(athlete != 12)
%>% select(-c(2:5))
scaleOWt1_hard1 <- scaleOW_Data %>% filter(measure == 2, day_class == 2) %>% filter(athlete != 12)
%>% select(-c(2:5))

#LOG MEASURES
#typical error between measures t1 on rest and mod day
te_RestModlogOW <- data.frame(mapply(function(x, y) te(log100(x), log100(y)), logOWt1_mod1,
logOWt1_rest1)) %>% rownames_to_column() %>% set_names(c("variable", "te_RestMod"))
te_RestModlogOW

#typical error between measures t1 on mod and hard day
te_ModHardlogOW <- data.frame(mapply(function(x, y) te(log100(x), log100(y)), logOWt1_hard1,
logOWt1_mod1)) %>% rownames_to_column() %>% set_names(c("variable", "te_ModHard"))
te_ModHardlogOW

#typical error between measures t1 on rest and hard day
te_RestHardlogOW <- data.frame(mapply(function(x, y) te(log100(x), log100(y)), logOWt1_hard1,
logOWt1_rest1)) %>% rownames_to_column() %>% set_names(c("variable", "te_RestHard"))
te_RestHardlogOW

#get overall TE TE/CV between rest, mod and easy three days
te_RestModHardlogOW <- bind_cols(te_RestModlogOW, te_ModHardlogOW, te_RestHardlogOW)
%>%
  select(c(variable, te_RestMod, te_ModHard, te_RestHard)) %>%
  mutate(te_Overall =
sqrt(((te_RestMod*te_RestMod*df_rest)+(te_ModHard*te_ModHard*df_rest)+(te_RestHard*te_RestHar
d*df_rest))/(df_rest+df_rest+df_rest))) %>% #keep df_rest as excluded athete 12
  select(-c(2:4))

#get 95% CI for te
low95CI_LogRestModHard <- data.frame(lower95CIFun_chi(df_Restmean,
```

```
te_RestModHardlogOW$te_Overall))
up95CI_LogRestModHard <- data.frame(upper95CIFun_chi(df_Restmean,
te_RestModHardlogOW$te_Overall))

#combine with typical error
te_RestModHardlogOW1 <- bind_cols(te_RestModHardlogOW, low95CI_LogRestModHard,
up95CI_LogRestModHard) %>% set_names(c("variable", "te", "te_lowCI", "te_upCI"))

#create overall reliability table with cv for Log variables
#kept df and n same as trials are on seperate days
RestModHardLogOW_table <- te_RestModHardlogOW1 %>%
  mutate(cv_RestModHard = 100*exp(te/100)-100,
         cv_lowCI = 100*exp(te_lowCI/100)-100,
         cv_upCI = 100*exp(te_upCI/100)-100) %>%
  select(-c(2:4))

#create signal to noise ratio
logSNR_table <- bind_cols(RestModHardLogOW_table[c(2:15),], OverallLogOW_table) %>%
  select(variable, cv_RestModHard, cv_lowCI, cv_upCI, cv_over, cv_lowCI1, cv_upCI1) %>%
  mutate(SNR = cv_RestModHard/cv_over,
         interpret1 = ifelse(cv_RestModHard>cv_upCI1, "good", ""),
         interpet2 = ifelse(cv_over<cv_lowCI, "good", ""))

logSNR_table
View(logSNR_table)

#SCALE MEASURES
#typical error between measures t1 on rest and mod day
te_RestModscaleOW <- data.frame(mapply(function(x, y) te(x, y), scaleOWt1_mod1, scaleOWt1_rest1))
%>% rownames_to_column() %>% set_names(c("variable", "te_RestMod"))
te_RestModscaleOW

#typical error between measures t1 on mod and hard day
te_ModHardscaleOW <- data.frame(mapply(function(x, y) te(x, y), scaleOWt1_hard1,
scaleOWt1_mod1)) %>% rownames_to_column() %>% set_names(c("variable", "te_ModHard"))
te_ModHardscaleOW

#typical error between measures t1 on rest and hard day
te_RestHardscaleOW <- data.frame(mapply(function(x, y) te(x, y), scaleOWt1_hard1, scaleOWt1_rest1))
%>% rownames_to_column() %>% set_names(c("variable", "te_RestHard"))
te_RestHardscaleOW

#get overall TE TE/CV between rest, mod and easy three days
```

```
te_RestModHardscaleOW <- bind_cols(te_RestModscaleOW, te_ModHardscaleOW, te_RestHardscaleOW) %>%
  select(c(variable, te_RestMod, te_ModHard, te_RestHard)) %>%
  mutate(te_Overall = sqrt(((te_RestMod*te_RestMod*df_rest)+(te_ModHard*te_ModHard*df_rest)+(te_RestHard*te_RestHard*df_rest))/(df_rest+df_rest+df_rest))) %>% #keep df_rest as excluded athete 12
  select(-c(2:4))

#get 95% CI for te
low95CI_ScaleRestModHard <- data.frame(lower95CIFun_chi(df_Restmean, te_RestModHardscaleOW$te_Overall))
up95CI_ScaleRestModHard <- data.frame(upper95CIFun_chi(df_Restmean, te_RestModHardscaleOW$te_Overall))

#combine with typical error
te_RestModHardscaleOW1 <- bind_cols(te_RestModHardscaleOW, low95CI_ScaleRestModHard, up95CI_ScaleRestModHard) %>% set_names(c("variable", "te", "te_lowCI", "te_upCI"))

#create signal to noise ratio
scaleSNR_table <- bind_cols(te_RestModHardscaleOW1[c(2:9),], OverallScaleOW_table) %>%
  select(variable, te, te_lowCI, te_upCI, te1, te_lowCI1, te_upCI1) %>%
  mutate(SNR = te/te1,
         interpret1 = ifelse(te>te_upCI1, "good", ""),
         interpet2 = ifelse(te1<te_lowCI, "good", ""))

scaleSNR_table
View(scaleSNR_table)

########################################
#COMPARE LOCATION MEASURES
logloc_table <- bind_cols(locLogOW_table, OverallLogOW_table) %>%
  select(variable, cv_loc, cv_lowCI, cv_upCI, cv_over, icc_loc, icc_lowCI, icc_highCI, stand_te, stand_telowCI, stand_teupCI) %>%
  mutate(cv_diff = cv_loc-cv_over)

View(logloc_table)
#get mean difference in CV between different locations
mean_diffLoclog <- mean(logloc_table$cv_diff)

#COMPARE LOCATION MEASURES
scaleloc_table <- bind_cols(locScaleOW_table, OverallScaleOW_table) %>%
  select(variable, te, te_lowCI, te_upCI, te1, icc_loc, icc_lowCI, icc_highCI, stand_te, stand_telowCI, stand_teupCI) %>%
```

```
  mutate(te_diff = te-te1)

scaleloc_table
View(scaleloc_table)
#get mean difference in TE between different locations
mean_diffLocscale <- mean(scaleloc_table$te_diff)
```

© 2020 by the authors. Submitted for possible open access publication under the terms and conditions of the Creative Commons Attribution (CC BY) license (http://creativecommons.org/licenses/by/4.0/).
